# The cost‐effectiveness of prophylaxis strategies for individuals with advanced HIV starting treatment in Africa

**DOI:** 10.1002/jia2.25469

**Published:** 2020-03-27

**Authors:** Simon M Walker, Edward Cox, Paul Revill, Victor Musiime, Mutsa Bwakura‐Dangarembizi, Jane Mallewa, Priscilla Cheruiyot, Kathryn Maitland, Nathan Ford, Diana M Gibb, A Sarah Walker, Marta Soares, the REALITY trial team, P Mugyenyi, C Kityo, V Musiime, P Wavamunno, E Nambi, P Ocitti, M Ndigendawani, S Kabahenda, M Kemigisa, J Acen, D Olebo, G Mpamize, A Amone, D Okweny, A Mbonye, F Nambaziira, A Rweyora, M Kangah, V Kabaswahili, J Abach, G Abongomera, J Omongin, I Aciro, A Philliam, B Arach, E Ocung, G Amone, P Miles, C Adong, C Tumsuiime, P Kidega, B Otto, F Apio, K Baleeta, A Mukuye, M Abwola, F Ssennono, D Baliruno, S Tuhirwe, R Namisi, F Kigongo, D Kikyonkyo, F Mushahara, D Okweny, J Tusiime, A Musiime, A Nankya, D Atwongyeire, S Sirikye, S Mula, N Noowe, A Lugemwa, M Kasozi, S Mwebe, L Atwine, T Senkindu, T Natuhurira, C Katemba, E Ninsiima, M Acaku, J Kyomuhangi, R Ankunda, D Tukwasibwe, L Ayesiga, J Hakim, K Nathoo, M Bwakura‐Dangarembizi, A Reid, E Chidziva, T Mhute, GC Tinago, J Bhiri, S Mudzingwa, M Phiri, J Steamer, R Nhema, C Warambwa, G Musoro, S Mutsai, B Nemasango, C Moyo, S Chitongo, K Rashirai, S Vhembo, B Mlambo, S Nkomani, B Ndemera, M Willard, C Berejena, Y Musodza, P Matiza, B Mudenge, V Guti, A Etyang, C Agutu, J Berkley, K Maitland, P Njuguna, S Mwaringa, T Etyang, K Awuondo, S Wale, J Shangala, J Kithunga, S Mwarumba, S Said Maitha, R Mutai, M Lozi Lewa, G Mwambingu, A Mwanzu, C Kalama, H Latham, J Shikuku, A Fondo, A Njogu, C Khadenge, B Mwakisha, A Siika, K Wools‐Kaloustian, W Nyandiko, P Cheruiyot, A Sudoi, S Wachira, B Meli, M Karoney, A Nzioka, M Tanui, M Mokaya, W Ekiru, C Mboya, D Mwimali, C Mengich, J Choge, W Injera, K Njenga, S Cherutich, M Anyango Orido, G Omondi Lwande, P Rutto, A Mudogo, I Kutto, A Shali, L Jaika, H Jerotich, M Pierre, J Mallewa, S Kaunda, J Van Oosterhout, B O'Hare, R Heydermann, C Gonzalez, N Dzabala, C Kelly, B Denis, G Selemani, L Nyondo Mipando, E Chirwa, P Banda, L Mvula, H Msuku, M Ziwoya, Y Manda, S Nicholas, C Masesa, T Mwalukomo, L Makhaza, I Sheha, J Bwanali, M Limbuni, D Gibb, M Thomason, AS Walker, S Pett, A Szubert, A Griffiths, H Wilkes, C Rajapakse, M Spyer, A Prendergast, N Klein, M Rauchenberger, N Van Looy, E Little, K Fairbrother, F Cowan, J Seeley, S Bernays, R Kawuma, Z Mupambireyi, F Kyomuhendo, S Nakalanzi, J Peshu, S Ndaa, J Chabuka, N Mkandawire, L Matandika, C Kapuya, I Weller, E Malianga, C Mwansambo, F Miiro, P Elyanu, E Bukusi, E Katabira, O Mugurungi, D Gibb, J Hakim, A Etyang, P Mugyenyi, J Mallewa, T Peto, P Musoke, J Matenga, S Phiri, H Lyall, V Johnston, F Fitzgerald, F Post, F Ssali, A Prendergast, A Arenas‐Pinto, A Turkova, A Bamford

**Affiliations:** ^1^ Centre for Health Economics University of York York UK; ^2^ Joint Clinical Research Centre Kampala Uganda; ^3^ University of Zimbabwe Clinical Research Centre Harare Zimbabwe; ^4^ College of Medicine University of Malawi and Malawi‐Liverpool‐Wellcome Trust Clinical Research Programme Blantyre Malawi; ^5^ Moi University School of Medicine Eldoret Kenya; ^6^ KEMRI Wellcome Trust Research Programme Kilifi Kenya; ^7^ Department of Infectious Diseases Imperial College London UK; ^8^ HIV/AIDS Department and Global Hepatitis Programme World Health Organization Geneva Switzerland; ^9^ MRC Clinical Trials Unit UCL London UK

**Keywords:** HIV, prophylaxis, fluconazole, late‐presenters, cost‐effectiveness

## Abstract

**Introduction:**

Many HIV‐positive individuals in Africa have advanced disease when initiating antiretroviral therapy (ART) so have high risks of opportunistic infections and death. The REALITY trial found that an enhanced‐prophylaxis package including fluconazole reduced mortality by 27% in individuals starting ART with CD4 <100 cells/mm^3^. We investigated the cost‐effectiveness of this enhanced‐prophylaxis package versus other strategies, including using cryptococcal antigen (CrAg) testing, in individuals with CD4 <200 cells/mm^3^ or <100 cells/mm^3^ at ART initiation and all individuals regardless of CD4 count.

**Methods:**

The REALITY trial enrolled from June 2013 to April 2015. A decision‐analytic model was developed to estimate the cost‐effectiveness of six management strategies in individuals initiating ART in the REALITY trial countries. Strategies included standard‐prophylaxis, enhanced‐prophylaxis, standard‐prophylaxis with fluconazole; and three CrAg testing strategies, the first stratifying individuals to enhanced‐prophylaxis (CrAg‐positive) or standard‐prophylaxis (CrAg‐negative), the second to enhanced‐prophylaxis (CrAg‐positive) or enhanced‐prophylaxis without fluconazole (CrAg‐negative) and the third to standard‐prophylaxis with fluconazole (CrAg‐positive) or without fluconazole (CrAg‐negative). The model estimated costs, life‐years and quality‐adjusted life‐years (QALY) over 48 weeks using three competing mortality risks: cryptococcal meningitis; tuberculosis, serious bacterial infection or other known cause; and unknown cause.

**Results:**

Enhanced‐prophylaxis was cost‐effective at cost‐effectiveness thresholds of US$300 and US$500 per QALY with an incremental cost‐effectiveness ratio (ICER) of US$157 per QALY in the CD4 <200 cells/mm^3^ population providing enhanced‐prophylaxis components are sourced at lowest available prices. The ICER reduced in more severely immunosuppressed individuals (US$113 per QALY in the CD4 <100 cells/mm^3^ population) and increased in all individuals regardless of CD4 count (US$722 per QALY). Results were sensitive to prices of the enhanced‐prophylaxis components. Enhanced‐prophylaxis was more effective and less costly than all CrAg testing strategies as enhanced‐prophylaxis still conveyed health gains in CrAg‐negative patients and savings from targeting prophylaxis based on CrAg status did not compensate for costs of CrAg testing. CrAg testing strategies did not become cost‐effective unless the price of CrAg testing fell below US$2.30.

**Conclusions:**

The REALITY enhanced‐prophylaxis package in individuals with advanced HIV starting ART reduces morbidity and mortality, is practical to administer and is cost‐effective. Efforts should continue to ensure that components are accessed at lowest available prices.

## 1 Introduction

In low‐ and middle‐income settings, more than a third of HIV‐positive individuals starting antiretroviral therapy (ART) present with advanced disease (CD4 ≤ 200 cells/mm^3^); over half of these have CD4 ≤ 100 cells/mm^3^ 1, 2. Such “late‐presenters” have increased risks of opportunistic infections and death shortly after starting ART 1, 3, particularly from severe bacterial infections 4, tuberculosis 5, 6, 7 and cryptococcal meningitis 8, 9. Prophylaxis, immediately before or concomitantly with ART, can prevent infections and reduce mortality 10.

The REALITY trial assessed the effectiveness of an “enhanced‐prophylaxis package” for HIV‐positive adults, adolescents and children greater than five years initiating ART with CD4 <100 cells/mm^3^. The package included a daily fixed‐dose combination tablet (FDC) of trimethoprim‐sulfmethoxazole, isoniazid and pyridoxine, 12 weeks’ fluconazole 100 mg daily, five days’ azithromycin 500 mg daily and a single dose of albendazole 10. Patients with clinical evidence of opportunistic infections at enrolment (e.g. cryptococcal meningitis) received infection‐specific treatment; cryptococcal antigen (CrAg) testing was not routinely performed. The enhanced‐prophylaxis package reduced all‐cause mortality by 27% (from 12.2% to 8.9%) over 24 weeks, significantly reducing cryptococcal deaths and deaths from unknown causes.

The 2017 Guideline Development Group at the World Health Organization (WHO) expressed concerns about the universal use of fluconazole in this population relating to costs, the potential for anti‐fungal resistance, and foetal safety among women of childbearing age. Hence, they recommended a reduced package of targeted prophylaxis including fluconazole as pre‐emptive treatment (not prophylaxis) only in individuals with CD4 <100 cells/mm^3^ who test positive for CrAg, consistent with WHO guidelines for cryptococcal meningitis at the time 11. Updated WHO guidance in 2018 recommended unrestricted fluconazole primary prophylaxis for individuals with CD4 <100 cells/mm^3^ (with consideration if CD4 <200 cells/mm^3^) where access to CrAg testing is limited, or where prolonged delays in receiving test results might occur, for example, ART initiation at lower level facilities (conditional recommendation; moderate‐certainty evidence).

We consider the cost‐effectiveness of universal enhanced‐prophylaxis versus other strategies including restricting fluconazole (as pre‐emptive treatment) to CrAg‐positive individuals, to identify the optimal strategy in individuals presenting with advanced HIV, and all newly presenting individuals, in the REALITY trial countries (Zimbabwe, Uganda, Malawi and Kenya).

## 2 Methods

A decision‐analytic model was developed to estimate costs and health outcomes of different strategies. Costs were estimated from a healthcare system perspective using country‐specific unit costs (price year 2016 US$). Health outcomes were expressed as life‐years and quality‐adjusted life‐years (QALYs) (a generic health measure capturing both quantity and quality of life; one QALY represents a year in perfect health). A 48‐week time horizon was used, reflecting trial follow‐up 10. Cost‐effectiveness was assessed using incremental cost‐effectiveness ratios (ICERs) and incremental net health benefits. ICERs represent the cost per additional unit of benefit of a strategy versus the next best strategy, where extendedly dominated strategies (i.e. those which cost more per additional unit of benefit than the next most effective strategy) are excluded from comparison. Incremental net health benefits (measured in QALYs) reflect the difference between the health generated with the strategy (vs. a reference strategy) and the health which would have been generated elsewhere if the required resources were used for alternative purposes (e.g. health generated by other treatments). Cost‐effectiveness thresholds represent the marginal productivity of the healthcare system (i.e. how much health would be generated elsewhere for a given resource), and we here considered commonly used threshold values of US$100, US$300 and US$500/QALY 12. At a given threshold, the strategy with the highest ICER below that threshold or with the highest incremental net health benefit is cost‐effective.

### 2.1 Patient population

Three populations of previously untreated HIV‐positive adults/adolescents with advanced HIV disease without evidence of clinical opportunistic infection (including Cryptococcus) at presentation in sub‐Saharan Africa were considered, with: (i) CD4 <200 cells/mm^3^; (ii) CD4 <100 cells/mm^3^ (REALITY inclusion criteria; 40 (2.2%) children aged 5‐12 years also recruited); and (iii) all individuals, regardless of CD4 count.

### 2.2 Management strategies

Six alternative management strategies over the first 12 weeks on ART were considered:

Strategy‐1: Standard‐prophylaxis: 12 weeks’ 160 mg trimethoprim/800 mg sulfamethoxazole daily (REALITY control group).
Strategy‐2: Enhanced‐prophylaxis: 12 weeks’ FDC of trimethoprim‐sulfmethoxazole, isoniazid (300 mg) and pyridoxine (25 mg) plus fluconazole (100 mg daily). Additionally, at ART initiation, single‐dose albendazole (400 mg) and five days’ azithromycin (500 mg daily) (REALITY intervention group).
Strategy‐3: 12 weeks’ trimethoprim/sulfamethoxazole and fluconazole (Strategy‐2 excluding isoniazid/pyridoxine).
Strategy‐4: First CrAg testing strategy: CrAg test at presentation, with CrAg‐positives receiving full enhanced‐prophylaxis (12 weeks’ FDC plus fluconazole (100 mg daily), single‐dose albendazole and five days azithromycin (Strategy‐2)) and CrAg‐negatives receiving standard‐prophylaxis (12 weeks’ trimethoprim/sulfamethoxazole (Strategy‐1)).
Strategy‐5: Second CrAg testing strategy: CrAg test at presentation, with CrAg‐positives receiving full enhanced‐prophylaxis (12 weeks’ FDC plus fluconazole (100 mg daily), single‐dose albendazole and five days azithromycin (Strategy‐2)) and CrAg‐negatives receiving 12 weeks’ FDC, single‐dose albendazole and five days azithromycin (Strategy‐2 excluding fluconazole).
Strategy‐6: Third CrAg testing strategy: CrAg test at presentation, with CrAg‐positives receiving 12 weeks’ trimethoprim/sulfamethoxazole plus fluconazole (100 mg daily) (Strategy‐3) and CrAg‐negatives receiving 12 weeks’ trimethoprim/sulfamethoxazole (Strategy‐1).

For all strategies, in countries where isoniazid prophylaxis became standard‐of–care during the trial (Uganda, Zimbabwe, Kenya), from 12 weeks onwards individuals in the trial received FDC, and in countries where isoniazid prophylaxis was not standard‐of‐care during the trial (Malawi), from 12 weeks onwards individuals received trimethoprim‐sulfamethoxazole alone. Event rates were very low after 12 weeks on ART 13, so differences in whether or not isoniazid/pyridoxine was part of standard‐prophylaxis after 12 weeks would have minimal effect. Full details of the strategies are provided in Table A1 in the Appendix.

### 2.3 Decision‐analytic model

The model is a Markov cohort model with one alive state and mortality from three competing risks: cryptococcal meningitis; tuberculosis, serious bacterial infection or other known cause; and unknown cause (Appendix Figure A1). The model has a one‐week cycle length and considers 48 cycles. During each one‐week cycle, individuals can die from one of the three competing risks or survive and incur costs based on the six resource categories (initial CrAg test, prophylaxis, ART, other concomitant medications, clinic visits and hospitalizations (cryptococcus‐specific and other)) and accrue life‐years and QALYs based on their health‐related quality‐of‐life (HRQoL) weight. Costs, life‐years and QALYs are then aggregated over the 48 weeks to estimate totals over the time period. The model was developed in Microsoft Excel.

The decision‐analytic model was evaluated separately over 10 cell/mm^3^ subgroups of baseline CD4 (i.e. 0‐10, 10‐20 cells/mm^3^, etc) as the relationships with CD4 are non‐linear and disaggregating into subgroups results in more accurate estimations. Estimates then weighted the 10‐cell subgroup results by the proportions in each subgroup within the population of interest. Individuals with CD4 >200 cells/mm^3^ were assumed to incur costs of prophylaxis and CrAg testing but receive no mortality benefit or impacts on other cost categories from prophylaxis. CD4 testing costs were not included but the value of CD4 testing for stratifying individuals to different strategies was considered as a scenario analysis. Table 1 indicates the sources of model inputs.

**Table 1:** Model inputs

Model inputs	Full details	Main source
Patient level covariates	Table A1	REALITY trial 10
Baseline CD4 distribution	Table A2	REALITY trial 10 DART trial 15 Carmona *et al*. 1
Cause specific mortality^a^	Table A4	REALITY trial 10
HRQoL	Table A7	REALITY trial 10 Jelsma *et al*. 19
Prophylaxis drug unit costs	Table 2	Pharmacies involved in REALITY trial
Other unit costs	Table A3	Economic Anlysis and Evaluation Team, WHO 16 International drug price indicator guide 17
Clinic visit costs	Table A5	REALITY trial 10 Economic Anlysis and Evaluation Team, WHO 16
ART costs	Table A5	REALITY trial 10 International drug price indicator guide 17
Hospitalization costs (cryptococcal and other)	Table A6	REALITY trial 10 Economic Anlysis and Evaluation Team, WHO 16
Concomitant drug costs	Table A6	REALITY trial 10 International drug price indicator guide 17

ART, antiretroviral therapy; HRQoL, health‐related quality‐of‐life.

^a^ DART trial used to inform suitability of extrapolation of results above CD4 100 cells/mm^3^.

### 2.4 Data

The main primary data source was REALITY which enrolled individuals from June 2013 through April 2015 10. These data informed participant characteristics (including proportions in CD4 subgroups <100 cells/mm^3^) (Appendix Tables A2 and A3), and were used to estimate cause‐specific mortality hazards over time, costs incurred and HRQoL weights, using regression analyses. Data from retrospective CrAg testing of stored samples from REALITY were also used 14. The DART trial 15 informed proportions in CD4 subgroups between 100 and 200 cells/mm^3^. A South African study informed the proportion of individuals presenting for ART with CD4 0‐100, 100‐200 and 200+ cells/mm^3^ (Appendix Table A3) 1.

### 2.5 Ethics and consent

The REALITY trial was approved by ethics committees in Zimbabwe, Uganda, Malawi, Kenya and the United Kingdom. Adult patients and guardians provided written informed consent with older children providing additional assent according to national guidelines.

### 2.6 Analysis

#### 2.6.1 Survival

Cause‐specific hazards for death from (i) cryptococcal meningitis, (ii) tuberculosis, serious bacterial infection or other known cause and (iii) unknown cause (Appendix Figure A1) were estimated from REALITY using competing risk piecewise exponential models (intervals 0‐8, 8‐24 and 24‐48 weeks reflecting changing hazards over time). Covariates considered for inclusion were: baseline CD4 (either absolute or log‐transformed), randomized treatment (standard‐prophylaxis vs. enhanced‐prophylaxis), baseline CrAg status, and an interaction between treatment and CrAg status. Covariates were included based on multivariable *p* < 0.1. Given the aim to extrapolate relationships to CD4 >100 cells/mm^3^, the choice of CD4 scale was also informed by DART data (baseline CD4 0‐200 cells/mm^3^) 15. On this basis, absolute scale CD4 parameterization was used for all analyses.

#### 2.6.2 Costs and resource use

Prophylaxis costs were based on the mean number of recorded prescriptions in REALITY and the minimum price for each drug across countries provided by pharmacies involved in REALITY (Table 2). The CrAg test cost was US$5.66 based on costs within REALITY (including discounted test, consumables and labour) 14. All inferences were based on REALITY data with unit costs taken from published sources (Appendix Table A4) 16, 17. ART and clinic visits costs were estimated using a panel data approach with weekly waves (48 waves). For concomitant medications, cryptococcal‐specific hospitalizations and other hospitalizations, a two‐stage regression approach was used; firstly (panel data) estimating the probability of a resource being used in any given week, and secondly regression estimating the mean weekly cost given the resource was used. Covariates considered for inclusion were randomized prophylaxis (enhanced‐prophylaxis vs. standard‐prophylaxis), baseline CD4, CrAg status, CrAg and treatment interaction, age, sex, time since ART initiation and three time to death indicators (subsequently dying within 0‐4, 5‐8 and 9‐12 weeks) reflecting potential resource implications of being close to death. Covariates were included if multivariable *p* < 0.1.

**Table 2:** Prophylaxis drug costs (2016 US$)

Costs	Zimbabwe	Uganda	Malawi	Kenya	Cheapest^a^
Albendazole (400 mg)	1.000	0.100	0.290	0.040	0.040
Azithromycin (500 mg)	4.470	0.264	0.562	0.156	0.156
Fluconazole (100 mg)	0.086	0.051	0.330	0.031	0.031
FDC^b^	0.038	0.057	0.026	0.054	0.026
Co‐trimoxazole (800/160 mg)	0.009	0.009	0.009	0.009	0.009
Isoniazid and pyridoxine (300/25 mg)	0.029	0.048	0.017	0.045	0.017
48‐week protocol prophylaxis medication costs					
Enhanced‐ prophylaxis	43.34	24.86	39.56	21.57	12.16
Standard‐ prophylaxis	10.33	15.12	3.02	14.36	6.70

Costs based on those reported by pharmacists involved in the REALITY trial.

FDC, fixed‐dose combination.

^a^ Base‐case prophylaxis drug costs: the lowest value taken across all country‐specific prophylaxis drug costs

^b^ Fixed dose combination of trimethoprim‐sulfmethoxazole (800/160 mg), isoniazid (300 mg) and pyridoxine (25 mg).

#### 2.6.3 Health‐Related Quality‐of‐Life

HRQoL was measured within REALITY using the EQ‐5D‐3L tool 18, a generic preference‐based measure of health encompassing five dimensions, at baseline, 2, 4, 8, 12, 18, 24, 36 and 48 weeks. HRQoL weights were derived from EQ‐5D‐3L responses using a published tariff from Zimbabwe 19, with 1 representing perfect health and 0 death. HRQoL weights were modelled using linear random effects regression with the following covariates: randomized prophylaxis, baseline CD4, age, sex, time since ART initiation and time to death indicators as above. Covariates were retained if multivariable *p* < 0.1. For an alternative scenario, baseline CD4 was replaced with baseline HRQoL score. Another scenario imposed a maximum HRQoL score of 0.9 for individuals in full health (to reflect the range of values in the original Zimbabwean study). QALYs were estimated from time alive and the HRQoL weights.

#### 2.6.4 Incorporating treatment effects

The decision model includes prophylaxis strategies that were not investigated within REALITY, specifically removing fluconazole from enhanced‐prophylaxis and adding fluconazole to standard‐prophylaxis. This requires assumptions about effects of fluconazole and other enhanced‐prophylaxis components. We assumed that differences between enhanced‐prophylaxis and standard‐prophylaxis on cryptococcal meningitis (mortality and hospitalization) were attributed to fluconazole only. Conversely, any effects of enhanced‐prophylaxis on other mortality causes or resource use were attributed to other parts of the package (Table 3).

**Table 3:** Breakdown of treatment effects by strategy

	Cryptococcal disease (mortality & hospitalizations)	Other hospitalizations	Deaths unknown causes
Effect in EP versus SP assumed due to	Fluconazole	Isoniazid, azithromycin and albendazole	Isoniazid, azithromycin and albendazole
No CrAg testing			
Strategy‐1: SP			
Strategy‐2: EP	X	X	X
Strategy‐3: SPplusF	X		
CrAg testing			
Strategy‐4: CrAg EP+ve SP−ve	X if CrAg‐positive	X if CrAg‐positive	X if CrAg‐positive
Strategy‐5: CrAg EP+ve EPlessF−ve	X if CrAg‐positive	X	X
Strategy‐6: CrAg SPplusF+ve SP−ve	X if CrAg‐positive		

X – receive treatment effect based on EP versus SP.

+ve, a positive CrAg test; −ve, a negative CrAg test; CrAg, cryptococcal antigen; EP, enhanced‐prophylaxis; EPlessF, enhanced‐prophylaxis less fluconazole; SP, standard‐prophylaxis; SPplusF, standard‐prophylaxis plus fluconazole.

#### 2.6.5 Probabilistic and scenario analyses

The probability of a strategy being cost‐effective was evaluated using probabilistic sensitivity analysis for the base‐case, with uncertainty in each of the equations used to populate the model incorporated assuming multivariate normality of the coefficients and propagated through the model using Monte Carlo simulation to determine overall decision uncertainty 20. Scenario analyses included alternative (including country‐specific) costs for prophylaxis drugs, US$12 annual FDC cost (based on ceiling price agreement and assumption of 50% reduction for bulk purchasing 21) and fluconazole cost at 200 mg dose (with no change in efficacy). We also considered an alternate model for cryptococcal mortality that included an interaction between enhanced‐prophylaxis and CrAg status. A final scenario considered WHO‐recommended fluconazole pre‐emptive treatment for CrAg‐positive individuals in CrAg testing strategies (two weeks’ 800 mg daily, then eight weeks’ 400 mg). There is no direct evidence on the effectiveness of this regimen (e.g. in the REMSTART trial 22 it was assessed in combination with adherence support and the effects of pre‐emptive fluconazole therefore cannot be isolated). We here assumed this regimen to remove all risk of cryptococcal mortality and hospitalization, which is an optimistic scenario given conflicting evidence on the impact on mortality 22, 23.

Finally, we determined the maximum price of CrAg tests at which a CrAg‐testing strategy would be cost‐effective and the maximum price of CD4 (to assign individuals to different strategies).

## 3 Results

We first present the results of the statistical analyses (on survival, costs and resource use and HRQoL) performed using the REALITY trial data, which will reflect outcomes of the trialled interventions, Strategy‐2 and Strategy‐1, in patients with CD4 <100. As described in the methods section, the results of these analyses were used to populate the decision model to evaluate the cost‐effectiveness of the six strategies.

### 3.1 Statistical analysis

#### 3.1.1 Survival

Enhanced‐prophylaxis (REALITY Intervention Group, Strategy‐2) significantly reduced cryptococcal and unknown mortality in relation to standard‐prophylaxis (REALITY Intervention Group, Strategy‐1): hazard ratios (HR) 0.30 [95% CI: 0.10, 0.92; *p* = 0.04] and 0.62 [0.40, 0.95; *p* = 0.03] respectively (Appendix Table A5). Although enhanced‐prophylaxis reduced morbidity, there was no evidence of effect on mortality from tuberculosis, serious bacterial infection or other known causes. CrAg‐positivity was only associated with increased cryptococcal mortality (HR = 42.62 [12.18, 149.22; *p* < 0.001]). Higher CD4 was associated with a decreased hazard for all three mortality causes. Mortality hazards decreased markedly over time after ART initiation. Estimated and observed competing risk survival curves were similar (Appendix Figure A2).

#### 3.1.2 Costs and resource use

Clinic visit costs were associated with age, country and time since ART initiation, and ART costs with age and country but not CD4 or CrAg status (Appendix Table A6). Enhanced‐prophylaxis reduced the probability of cryptococcal hospitalizations and other hospitalizations in relation to standard‐prophylaxis (odds ratio (OR) 0.56 [0.33, 0.94; *p* = 0.28] and 0.84 [0.67, 1.06; *p* = 0.14] respectively), while CrAg‐positivity was associated with increased probability of cryptococcal hospitalization (OR = 99.22 [44.65, 220.48; *p* < 0.001]) but reduced probability of other hospitalizations (OR = 0.44 [0.25, 0.76; *p* = 0.003]) (Appendix Table A7). Enhanced‐prophylaxis and CrAg‐positivity did not impact the probability of using concomitant drugs in any given cycle but did reduce and increase costs respectively if an individual did use concomitant drugs in that cycle (weekly costs difference of $−0.16 [−0.34, 0.02] and $1.20 [0.87, 1.53] respectively). The probability of each incurred cost increased in the weeks prior to death.

#### 3.1.3 HRQoL weights

Enhanced‐prophylaxis was associated with higher HRQoL weights than standard‐prophylaxis although this was not statistically significant (incremental HRQoL weight 0.007 [−0.002, 0.016; *p* = 0.12]) (Appendix Table A8). Time since ART initiation was also associated with higher HRQoL weights (i.e. HRQoL improved with time), and in the weeks prior to death HRQoL weights decreased.

### 3.2 Base‐case cost‐effectiveness

**Figure 1:**
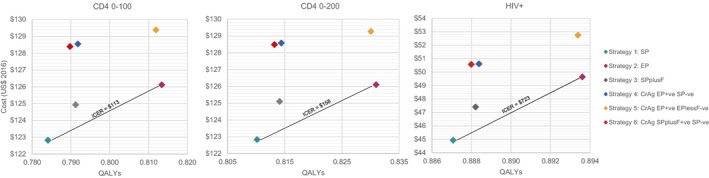
Cost‐effectiveness planes. EP, enhanced‐prophylaxis; EPlessF, enhanced‐prophylaxis less fluconazole; ICER, incremental cost‐effectiveness ratio; QAYL, quality‐adjusted life‐years; SP, standard prophylaxis; SPplusF, standard prophylaxis plus fluconazole.

In the CD4 <200 cells/mm^3^ population (Table 4, Figure 1; narrower CD4 subgroups in Appendix Table A9), standard‐prophylaxis (Strategy‐1) was the least costly and least effective strategy. Enhanced‐prophylaxis (Strategy‐2) was the third least costly, and also the most effective strategy, with an ICER of US$157/QALY versus standard‐prophylaxis (with standard‐prophylaxis plus fluconazole extendedly dominated). Enhanced‐prophylaxis was more effective and less costly than all CrAg testing strategies (Strategies 4‐6). At cost‐effectiveness thresholds of US$300 and US$500, enhanced‐prophylaxis is cost‐effective, with probability of not being cost‐effective of only 22% and 8% respectively; the incremental net health benefits per 1000 individuals are 9.91 and 14.26 QALYs respectively.

**Table 4:** Base‐case cost‐effectiveness results

CD4 <200 cells/mm^3^	Costs (US$)	QALY (max 0.923)	LY (max 0.923)	Incremental net health benefit (probability of being cost‐effective), QALY			Incremental cost‐effectiveness ratios
				*K* = $100	*K* = $300	*K* = $500	Cost per QALY
Strategy‐1: SP	$122.84	0.81015	0.85028	– (0.835)	– (0.217)	– (0.077)	–
Strategy‐3: SPplusF	$125.11	0.81408	0.85409	−0.01881 (0)	−0.00366 (0)	−0.00062 (0.001)	Ext dominated
Strategy‐2: EP	$126.10	0.83095	0.86451	−0.01186 (0.165)	0.00991 (0.783)	0.01426 (0.922)	$157.01
Strategy‐6: CrAg SPplusF+ve SP−ve	$128.49	0.81319	0.85323	−0.05346 (0)	−0.0158 (0)	−0.00826 (0)	Dominated
Strategy‐4: CrAg EP+ve SP−ve	$128.58	0.81438	0.85405	−0.05324 (0)	−0.01493 (0)	−0.00727 (0)	Dominated
Strategy‐5: CrAg EP+ve EPlessF−ve	$129.28	0.83004	0.86363	−0.04451 (0)	−0.00158 (0)	0.00701 (0)	Dominated
CD4 <100 cells/mm^3^							
Strategy‐1: SP	$122.83	0.78407	0.81875	– (0.677)	– (0.021)	– (0.005)	–
Strategy‐3: SPplusF	$124.94	0.79120	0.82568	−0.01396 (0)	0.0001 (0.001)	0.00292 (0.002)	Ext dominated
Strategy‐2: EP	$126.13	0.81339	0.84140	−0.00368 (0.322)	0.01832 (0.979)	0.02272 (0.993)	$112.53
Strategy‐6: CrAg SPplusF+ve SP−ve	$128.40	0.78974	0.82427	−0.05007 (0)	−0.01291 (0)	−0.00548 (0)	Dominated
Strategy‐4: CrAg EP+ve SP−ve	$128.56	0.79177	0.82573	−0.04959 (0)	−0.01139 (0)	−0.00376 (0)	Dominated
Strategy‐5: CrAg EP+ve EPlessF−ve	$129.39	0.81188	0.83994	−0.03781 (0)	0.00593 (0)	0.01468 (0)	Dominated
All individuals with HIV^a^							
Strategy‐1: SP	$44.93	0.88708	0.90027	–	–	–	–
Strategy‐3: SPplusF	$47.41	0.88821	0.90137	−0.02369	−0.00714	−0.00383	Ext dominated
Strategy‐2: EP	$49.65	0.89361	0.90464	−0.04067	−0.00920	−0.00291	$722.27
Strategy‐6: CrAg SPplusF+ve SP−ve	$50.58	0.88799	0.90116	−0.05558	−0.01792	−0.01038	Dominated
Strategy‐4: CrAg EP+ve SP−ve	$50.61	0.88838	0.90143	−0.05552	−0.01764	−0.01006	Dominated
Strategy‐5: CrAg EP+ve EPlessF−ve	$52.75	0.89339	0.90443	−0.07191	−0.01976	−0.00933	Dominated

CrAg, cryptococcal antigen; EP, enhanced‐prophylaxis; EPlessF, enhanced‐prophylaxis less fluconazole; Ext dominated, extendedly dominated; *K*, cost‐effectiveness threshold; LY, life years; QALY, quality adjusted life years; SP, standard‐prophylaxis; SPplusF, standard‐prophylaxis plus fluconazole.

^a^ In patients with CD4 >200 cell/mm^3^, only costs of the prophylaxis and CrAg testing are included.

Results were similar in the CD4 <100 cells/mm^3^ population (Table 4, Figure 1), with standard‐prophylaxis being the least costly and least effective strategy, and enhanced‐prophylaxis the third least costly, but most effective. Given higher mortality in this more advanced population, the ICER for enhanced‐prophylaxis versus standard‐prophylaxis was lower at US$113/QALY; therefore enhanced‐prophylaxis remains cost‐effective at the two higher cost‐effectiveness thresholds with lower decision uncertainty.

Considering all individuals presenting with HIV, regardless of CD4 count (Table 4, Figure 1), the ICER for enhanced‐prophylaxis versus standard‐prophylaxis was US$722/QALY, indicating that it is not cost‐effective at any of the cost‐effectiveness thresholds considered.

The largest cost‐savings from enhanced‐prophylaxis relate to hospitalizations, and are not markedly different for clinic visits or other (non‐prophylaxis) drug costs (Appendix Figure A3).

### 3.3 Scenario analyses

Scenario analyses in the CD4 <200 cells/mm^3^ population (Table 5, other populations in Appendix Tables A10‐A12) show results are sensitive to the substantial between‐country differences in drug prices (Table 2). For Kenyan or Ugandan prices, results are similar to the base‐case but with higher ICERs for enhanced‐prophylaxis, US$229 and US$353/QALY respectively. For Malawian or Zimbabwean prices, results change as enhanced‐prophylaxis becomes the most expensive strategy, due to high local fluconazole and azithromycin costs respectively. Enhanced‐prophylaxis and the second CrAg testing strategy (no longer dominated in this scenario) present ICERs above all thresholds considered, hence standard‐prophylaxis is cost‐effective. Other scenarios are similar to the base‐case but with different ICERs for enhanced‐prophylaxis: US$178/QALY for FDC costing US$12 per year; US$292/QALY for 200 mg fluconazole prophylaxis; US$185/QALY truncating HRQoL at 0.9; and US$162/QALY for alternative model for cryptococcal meningitis mortality. Considering WHO‐recommended pre‐emptive fluconazole treatment in CrAg‐positive individuals, the second CrAg strategy was the most effective but also the most costly, with an ICER of US$4,509/QALY. The impact of the scenarios was broadly similar across the different populations, with results most sensitive to drug prices, and the cost‐effectiveness of enhanced‐prophylaxis more favourable in the most advanced population.

**Table 5:** Scenario analyses for the CD4 <200 cells/mm^3^ population

CD4 <200 cells/mm^3^	Costs (US$)	QALY	LY	Incremental net health benefit			Incremental cost‐effectiveness ratios
				*K* = $100	*K* = $300	*K* = $500	Cost per QALY
Country‐specific drug costs							
Strategy‐1: SP	$126.10	0.81361	0.85371				
Strategy‐6: CrAg SPplusF+ve SP−ve	$132.04	0.81640	0.85643	−0.05662	−0.01701	−0.00909	Ext dominated
Strategy‐4: CrAg EP+ve SP−ve	$132.69	0.81757	0.85722	−0.06198	−0.01802	−0.00922	Ext dominated
Strategy‐3: SPplusF	$133.50	0.81706	0.85707	−0.07051	−0.02121	−0.01134	Dominated
Strategy‐5: CrAg EP+ve EPlessF−ve	$142.05	0.83281	0.86635	−0.14034	−0.03398	−0.01271	$830.99
Strategy‐2: EP	$143.90	0.83349	0.86701	−0.10153	−0.02245	−0.00664	$2,726.13
Zimbabwe‐specific drug costs							
Strategy‐1: SP	$124.87	0.81361	0.85371				
Strategy‐6: CrAg SPplusF+ve SP−ve	$130.76	0.81640	0.85643	−0.05603	−0.01682	−0.00898	Ext dominated
Strategy‐3: SPplusF	$131.34	0.81706	0.85707	−0.06126	−0.01812	−0.00949	Ext dominated
Strategy‐4: CrAg EP+ve SP−ve	$132.40	0.81757	0.85722	−0.07131	−0.02113	−0.01109	Ext dominated
Strategy‐5: CrAg EP+ve EPlessF−ve	$157.48	0.83281	0.86635	−0.30683	−0.08948	−0.04601	Ext dominated
Strategy‐2: EP	$158.61	0.83349	0.86701	−0.26151	−0.07578	−0.03863	$1,697.51
Uganda‐specific drug costs							
Strategy‐1: SP	$126.87	0.81361	0.85371				
Strategy‐3: SPplusF	$130.49	0.81706	0.85707	−0.03282	−0.00864	−0.00381	Ext dominated
Strategy‐6: CrAg SPplusF+ve SP−ve	$132.59	0.81640	0.85643	−0.05445	−0.01629	−0.00866	Dominated
Strategy‐4: CrAg EP+ve SP−ve	$132.83	0.81757	0.85722	−0.05567	−0.01591	−0.00796	Ext dominated
Strategy‐2: EP	$133.88	0.83349	0.86701	−0.05022	−0.00349	0.00586	$352.65
Strategy‐5: CrAg EP+ve EPlessF−ve	$135.49	0.83281	0.86635	−0.03424	−0.00091	0.00576	Dominated
Malawi‐specific drug costs							
Strategy‐1: SP	$123.74	0.81361	0.85371				
Strategy‐6: CrAg SPplusF+ve SP−ve	$130.71	0.81640	0.85643	−0.06691	−0.02044	−0.01115	Ext dominated
Strategy‐4: CrAg EP+ve SP−ve	$131.04	0.81757	0.85722	−0.06899	−0.02035	−0.01063	Ext dominated
Strategy‐5: CrAg EP+ve EPlessF−ve	$133.45	0.83281	0.86635	−0.07792	−0.01317	−0.00023	$505.87
Strategy‐3: SPplusF	$148.96	0.81706	0.85707	−0.24874	−0.08062	−0.04699	Dominated
Strategy‐2: EP	$153.81	0.83349	0.86701	−0.21385	−0.05989	−0.02910	$30,009.37
Kenya‐specific drug costs							
Strategy‐1: SP	$126.42	0.81361	0.85371				
Strategy‐3: SPplusF	$128.68	0.81706	0.85707	−0.01906	−0.00406	−0.00106	Ext dominated
Strategy‐2: EP	$130.98	0.83349	0.86701	−0.02564	0.00470	0.01077	$229.00
Strategy‐6: CrAg SPplusF+ve SP−ve	$132.07	0.81640	0.85643	−0.05364	−0.01602	−0.00850	Dominated
Strategy‐4: CrAg EP+ve SP−ve	$132.24	0.81757	0.85722	−0.05422	−0.01543	−0.00767	Dominated
Strategy‐5: CrAg EP+ve EPlessF−ve	$134.17	0.83281	0.86635	−0.03917	−0.00255	0.00477	Dominated
FDC costs $12 per year							
Strategy‐1: SP	$123.76	0.81361	0.85371				
Strategy‐3: SPplusF	$125.12	0.81706	0.85707	−0.01017	−0.00109	0.00072	Ext dominated
Strategy‐2: EP	$127.31	0.83349	0.86701	−0.01559	0.00806	0.01278	$178.41
Strategy‐6: CrAg SPplusF+ve SP−ve	$129.34	0.81640	0.85643	−0.05304	−0.01582	−0.00838	Dominated
Strategy‐4: CrAg EP+ve SP−ve	$129.51	0.81757	0.85722	−0.05353	−0.01520	−0.00753	Dominated
Strategy‐5: CrAg EP+ve EPlessF−ve	$130.50	0.83281	0.86635	−0.03803	−0.00217	0.00500	Dominated
200 mg fluconazole dosage							
Strategy‐1: SP	$122.89	0.81361	0.85371				
Strategy‐3: SPplusF	$127.54	0.81706	0.85707	−0.04311	−0.01207	−0.00587	Ext dominated
Strategy‐6: CrAg SPplusF+ve SP−ve	$128.66	0.81640	0.85643	−0.05492	−0.01645	−0.00875	Dominated
Strategy‐2: EP	$128.68	0.83349	0.86701	−0.03807	0.00056	0.00829	$291.55
Strategy‐4: CrAg EP+ve SP−ve	$128.76	0.81757	0.85722	−0.05481	−0.01563	−0.00779	Dominated
Strategy‐5: CrAg EP+ve EPlessF−ve	$129.45	0.83281	0.86635	−0.00332	0.00940	0.01194	Dominated
Full‐health EQ‐5D score 0.9							
Strategy‐1: SP	$122.89	0.74263	0.85371				
Strategy‐3: SPplusF	$125.12	0.74573	0.85707	−0.01925	−0.00435	−0.00137	Ext dominated
Strategy‐2: EP	$126.10	0.76002	0.86701	−0.01478	0.00667	0.01096	$184.95
Strategy‐6: CrAg SPplusF+ve SP−ve	$128.52	0.74514	0.85643	−0.05379	−0.01626	−0.00875	Dominated
Strategy‐4: CrAg EP+ve SP−ve	$128.61	0.74617	0.85722	−0.05373	−0.01555	−0.00792	Dominated
Strategy‐5: CrAg EP+ve EPlessF−ve	$129.30	0.75941	0.86635	−0.02808	−0.00024	0.00533	Dominated
Alternate specification for CM survival							
Strategy‐1: SP	$122.89	0.81363	0.85373				
Strategy‐3: SPplusF	$125.12	0.81705	0.85706	−0.01888	−0.00401	−0.00104	Ext dominated
Strategy‐2: EP	$126.10	0.83348	0.86700	−0.01228	0.00914	0.01343	$161.85
Strategy‐6: CrAg SPplusF+ve SP−ve	$128.54	0.81664	0.85666	−0.05353	−0.01584	−0.00830	Dominated
Strategy‐4: CrAg EP+ve SP−ve	$128.64	0.81782	0.85746	−0.05333	−0.01498	−0.00731	Dominated
Strategy‐5: CrAg EP+ve EPlessF−ve	$129.33	0.83306	0.86659	−0.02606	0.00199	0.00760	Dominated
WHO pre‐emptive therapy|CrAg+ve							
Strategy‐1: SP	$122.89	0.81361	0.85371				
Strategy‐2: EP	$126.10	0.83349	0.86701	−0.01230	0.00915	0.01344	$161.88
CrAg SP & WHO+ve SP−ve	$128.61	0.81794	0.85791	−0.05289	−0.01475	−0.00712	Dominated
Strategy‐4: CrAg EP+ve SP−ve	$128.61	0.81757	0.85722	−0.05331	−0.01513	−0.00749	Dominated
CrAg EP & WHO+ve SP−ve	$128.63	0.81896	0.85856	−0.05205	−0.01378	−0.00613	Dominated
CrAg EP & WHO+ve EPlessF−ve	$129.31	0.83420	0.86769	−0.04366	−0.00083	0.00774	$4,509.19

CrAg, cryptococcal antigen; EP, enhanced‐prophylaxis; EPlessF, enhanced‐prophylaxis less fluconazole; Ext dominated, extendedly dominated; FDC, fixed dose combination; *K*, cost‐effectiveness threshold; LY, life years; QALY, quality adjusted life years; SP, standard‐prophylaxis; SPplusF, standard‐prophylaxis plus fluconazole; WHO, World Health Organization recommended fluconazole regimen.

The maximum CrAg test prices that make a CrAg testing strategy cost‐effective are markedly lower than the base‐case value of US$5.66 (Appendix Figure A4); at a US$300/QALY threshold, the maximum price should be US$2.26 in the CD4 <200 cells/mm^3^ population.

The maximum CD4 test price to stratify individuals to different strategies is US$2.21 at a threshold of US$500/QALY (Appendix Figure A4).

## 4 Discussion

This study is the first to compare enhanced‐prophylaxis packages for opportunistic infections with other prophylaxis strategies, including CrAg‐based targeted prophylaxis, for HIV‐positive individuals in sub‐Saharan Africa. The full enhanced‐prophylaxis package (tested in REALITY) confers significant health benefits and is cost‐effective at accepted cost‐effectiveness thresholds providing constituent drugs are available at cheapest prices in the four countries included in the REALITY trial. The incremental cost of prophylaxis per patient was only $5.46 versus standard care, which is offset by cost‐savings elsewhere. Enhanced‐prophylaxis was more effective and less costly than all CrAg testing strategies as the enhanced‐prophylaxis still conveyed health gains in CrAg‐negative patients and the savings from targeting prophylaxis based on CrAg status do not compensate for the cost of CrAg testing.

The cost‐effectiveness of enhanced‐prophylaxis was, however, highly sensitive to drug component prices, which varied widely by country. Using country specific costs, enhanced‐prophylaxis presented an ICER of US$230/QALY, US$353/QALY, and US$501/QALY in Kenya, Uganda and Malawi respectively. For enhanced‐prophylaxis to be recommended in late presenters, efforts are needed to minimize drug prices. Lessons should be learned from international efforts to negotiate and drive down prices of ART in sub‐Saharan Africa 24.

Our analyses indicate that strategies involving CrAg testing, as currently recommended by WHO guidelines for advanced HIV disease, are not cost‐effective at a CrAg test cost of US$5.66, the actual cost for the test within the REALITY trial. Sensitivity analysis indicate that, for the CD4 <200 cells/mm^3^ population, CrAg testing only becomes cost‐effective (using a cost‐effectiveness threshold of US$300/QALY) at a much lower cost of US$2.26 per test. Whilst the manufacturer quotes test costs of USD$2.50, this does not include shipping or labour. Other recent studies have estimated costs of USD$3.41‐$5.24 in Africa 25, 26, 27 while study partners have indicated costs of USD$7‐$10.

Our study also shows the value of stratifying individuals according to pre‐ART CD4 count, whose measurement has recently been reported to greatly reduce mortality 28. It is difficult to identify individuals with advanced HIV via symptoms alone; for example, all individuals in REALITY had CD4 <100 cells/mm^3^ (median 37 cells/mm^3^), but around half had few or no clinical symptoms. Using enhanced‐prophylaxis in all individuals at presentation is not cost‐effective; CD4 tests must cost under US$2.28 to be cost‐effective if solely used for enhanced‐prophylaxis stratification (at the US$500/QALY threshold), considerably below published CD4 prices 29. However, CD4 testing also identifies individuals at high risk of imminent morbidity/mortality after restarting ART after interruption, or switching to second‐line ART, where enhanced‐prophylaxis may also be valuable 11.

A previous study examining the cost‐effectiveness of CrAg screening to target prophylaxis in South Africa found the CrAg screening strategies to be more effective and less costly than universal fluconazole prophylaxis 30. However, the universal prophylaxis considered was 200 mg daily for a year, rather than 100 mg daily for 12 weeks as in REALITY, and the cost of fluconazole was much higher than in REALITY countries. The study also considered only cryptococcal mortality in CrAg‐positive individuals (i.e. assumed no benefits of fluconazole in individuals testing CrAg‐negative, despite the potential for false‐negatives and the possibility of infection with cryptococcus shortly after ART initiation 14). Additional benefits from targeted pre‐emptive treatment with higher fluconazole doses (as defined by WHO) were considered in our scenario analysis and the additional cost was not value‐for‐money even if it eliminated all cryptococcal mortality and hospitalizations. Importantly, while not considered explicitly here, enhanced‐prophylaxis can be administered immediately to individuals starting ART, whereas in some situations additional CrAg testing may result in delays in ART initiation with likely mortality in those at high risk awaiting test results.

The REMSTART trial investigated CrAg screening and community‐based early adherence support in individuals starting ART with CD4 <200 cells/mm^3^, with CrAg‐positive individuals receiving 10 weeks of fluconazole (at WHO recommended doses) in Tanzania and Zambia. The trial found that CrAg screening and adherence support was cost‐effective versus standard care in low‐income settings 22, 27. However, this analysis did not consider alternative prophylaxis strategies and importantly did not separate benefits of CrAg screening from adherence support in the intervention arm. A recent modelling study for Botswana found reflexive CrAg screening of individuals with CD4 <100 cells/mm^3^ and treatment of identified CrAg‐positives was cost‐effective with an incremental cost of US$2 per DALY averted versus no screening. 25 However, this study did not consider a universal prophylaxis strategy in populations with low CD4. Similarly, studies of national CrAg screening programmes in individuals with HIV in Uganda and Vietnam found they were a cost‐effective use of resources versus not screening 26, 31, but again did not consider a universal prophylaxis strategy and focussed on screening the whole HIV population, rather than those presenting with advanced HIV in whom we found universal prophylaxis to be cost‐effective.

Study strengths include the use of the best available data to assess the cost‐effectiveness of different prophylaxis strategies based on the REALITY trial package and CrAg screening, including factor‐specific models based on extensive detailed data collected in the trial. The model closely fits REALITY survival data suggesting good internal validity. Using a decision‐analytic modelling approach enabled us to consider cost‐effectiveness in individuals with CD4 100‐200 cells/mm^3^. Uncertainty in model parameters was reflected using probabilistic sensitivity analysis, and the impact on results of key uncertainties was quantified using scenario analysis.

Limitations include the fact that factors such as improved adherence in the trial may affect generalizability. However, comparisons between randomized groups (on which cost‐effectiveness is based) are not affected by this and higher underlying mortality rates outside of a trial would mean our results are conservative regarding benefits from enhanced‐prophylaxis. Some of our strategies were not considered directly in the trial, and analyses hence required assumptions about the impact of the individual package components on different reasons for mortality and hospitalization (these are highlighted in Table 3). Modelling cost‐effectiveness in subgroups not included in the REALITY trial (i.e. individuals with CD4 >100 cells/mm^3^) involved extrapolating relationships between CD4 and cause‐specific mortality hazards, costs and HRQoL. The trial, and our analyses, only considered 100 mg fluconazole daily prophylaxis; higher doses might have higher efficacy in CrAg‐positive individuals 32, although the lower REALITY dose reduced cryptococcal mortality. Our scenario analysis showed that even if higher pre‐emptive treatment doses eliminated all cryptococcal mortality and hospitalizations, it would not be cost‐effective at accepted thresholds. Another limitation is that hospitalization costs were obtained from WHO CHOICE and do not distinguish by reason for hospitalization or capture all treatments or diagnostic tests received, although drug costs would have been included as concomitant medications. This will underestimate cryptococcal hospitalization costs, which means our analyses will be conservative towards the extended‐prophylaxis regime.

Some potential benefits of prophylaxis are not included in the model, for example, reductions in oral and oesophageal candida with fluconazole, which impacts HRQoL, and the consequences of any delays in ART initiation while waiting for CrAg test results (recognized in WHO 2018 cryptococcal guidelines 33 which state that “fluconazole primary prophylaxis should be made available in settings in which CrAg screening is not available or there may be prolonged delays in receiving the result”). Both make the current analysis conservative with respect to enhanced‐prophylaxis.

Our analyses did not include effects of teratogenicity because fluconazole is classed as Category D only for doses ≥ 400 mg daily; assuming that no pregnant woman would start fluconazole under universal or targeted strategies, the probability of new pregnancy in 12 weeks is low, at least in the advanced population. Finally, the model only estimated results over 48 weeks, reflecting trial follow‐up. Absolute mortality benefits with enhanced‐prophylaxis versus standard‐prophylaxis are likely to persist much longer.

## 5 Conclusions

In conclusion, the REALITY enhanced‐prophylaxis package in individuals initiating ART with CD4 <200 cells/mm^3^ confers significant benefit to individuals and appears cost‐effective at accepted cost‐effectiveness thresholds. Efforts should continue to ensure that the components of the package can be accessed at lowest available prices.

## Competing interests

All authors excluding NF report grants from MRC/DfID/Welllcome Trust Joint Global Health Trials during the conduct of this study. Cipla Ltd, Gilead Sciences and Merck donated ART as part of the REALITY trial and Cipla Ltd also donated prophylaxis drugs.

## Authors' Contributions

EC, MS and SMW designed the cost‐effectiveness model and statistical analysis with input from DG, PR and ASW. EC and SMW implemented the cost‐effectiveness model and statistical analysis with input from DG, PR, MS and ASW. DG and ASW contributed to the designed and undertaking of the REALITY trial. MS and SMW drafted the manuscript with substantial input from EC, PR, VM, MBD, JM, PC, KM, NF, DG and ASW. SMW, EC, PR, VM, MBD, JM, PC, KM, NF, DMG, ASW and MS approved the submitted version of the manuscript.

## Supporting information


**Appendix S1. ** Supplementary Figures and Tables.
