# Supplementary material for: The cost‐effectiveness of prophylaxis strategies for individuals with advanced HIV starting treatment in Africa
Source: J Int AIDS Soc. 2020 Mar 27;23(3):e25469. doi: 10.1002/jia2.25469 (PMC7099175; doi:10.1002/jia2.25469)
Supplement: Supplementary file 1 — Appendix S1. Supplementary Figures and Tables. [file JIA2-23-e25469-s001.docx]

**Appendix**

Figure A1: Model structure


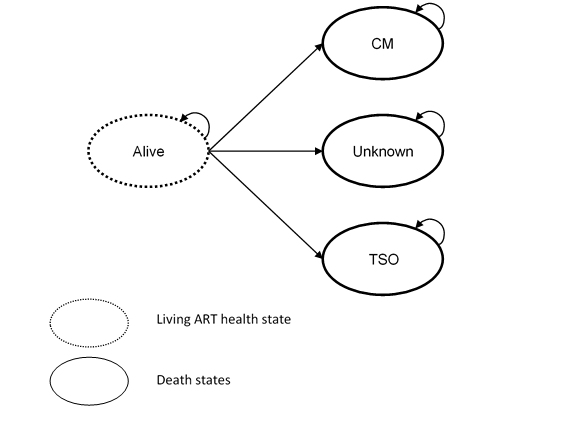


Note: CM: Cyptoccocal meningitis; TSO: Tuberculosis, serious bacterial infections and other known causes

Figure A2: Survival estimates based on the competing risk approach -REALITY trial


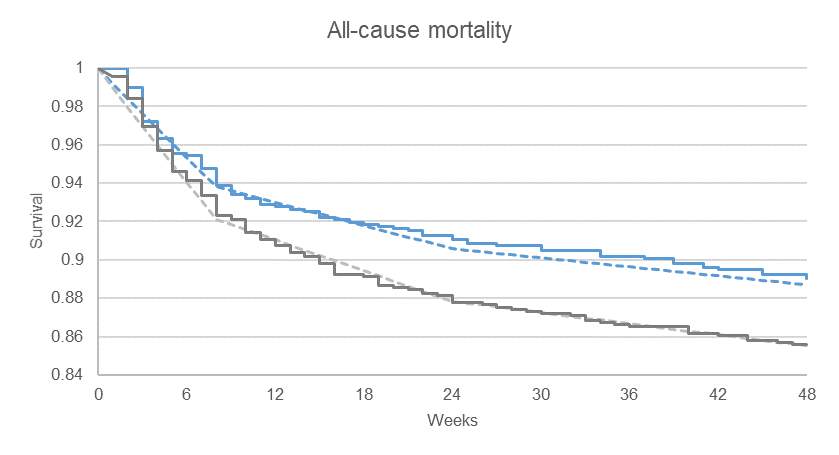

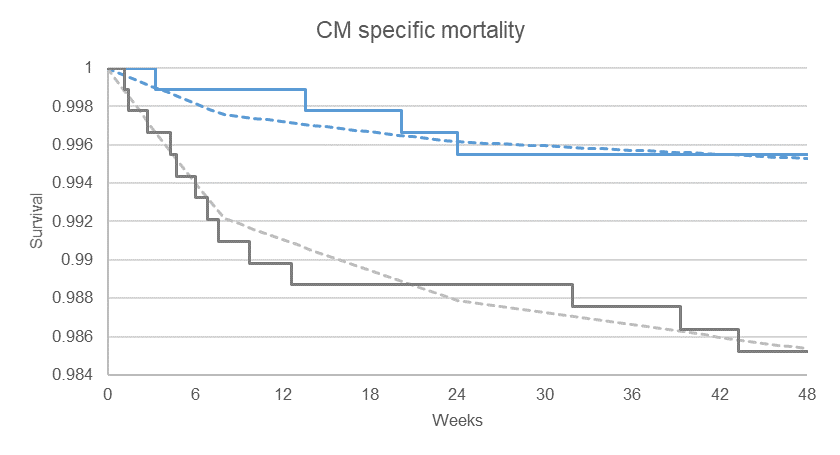

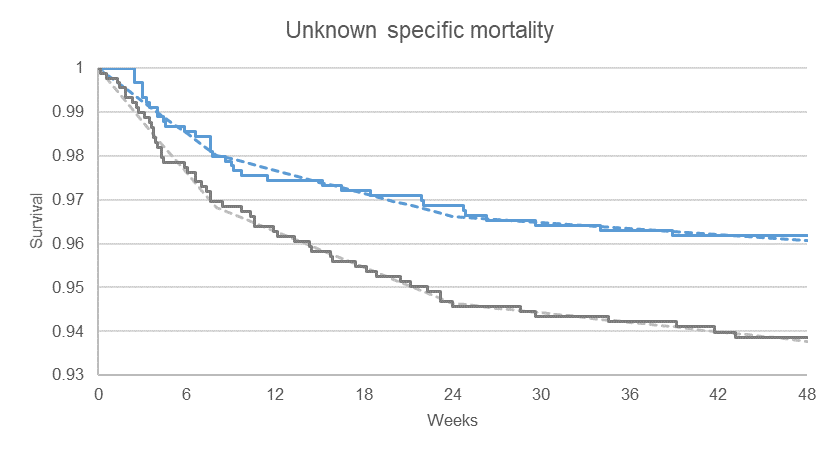

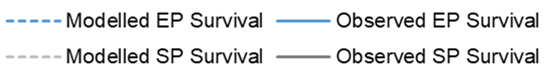

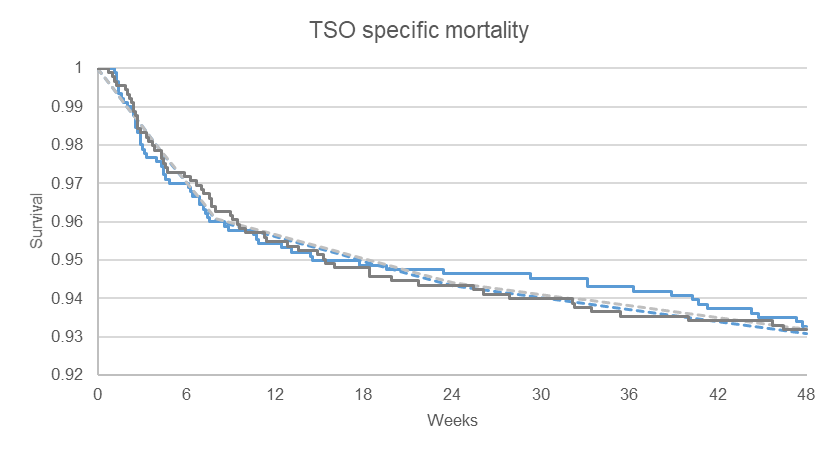


| Number at risk (number censored) | | | | | | | | |  |  |
| --- | --- | --- | --- | --- | --- | --- | --- | --- | --- | --- |
| SP | 899 (0) | 833 (16) | 798 (19) | 784 (19) | 768 (29) | 755 (31) | 748 (36) | 735 (41) | | 637 |
| EP | 906 (0) | 854 (11) | 827 (18) | 815 (18) | 807 (22) | 798 (23) | 794 (27) | 784 (28) | | 689 |

| Number at risk (number censored) | | | | | | | | | | | | | | | |  |  |  |  |
| --- | --- | --- | --- | --- | --- | --- | --- | --- | --- | --- | --- | --- | --- | --- | --- | --- | --- | --- | --- |
| SP | 899 (0) | | 833 (62) | | 798 (92) | | 784 (105) | | 768 (127) | | 755 (134) | | 748 (144) | | 735 (152) | | | 637 | |
| EP | 906 (0) | | 854 (51) | | 827 (82) | | 815 (89) | | 807 (98) | | 798 (104) | | 794 (111) | | 784 (118) | | | 689 | |
|  | |  | |  | |  | |  | |  |  |  | |  | | |  | |  |

| Number at risk (number censored) | | | | | | | |  |  |  |
| --- | --- | --- | --- | --- | --- | --- | --- | --- | --- | --- |
| SP | 899 (0) | 833 (47) | 798 (68) | 784 (75) | 768 (89) | 755 (94) | 748 (104) | | 735 (111) | 637 |
| EP | 906 (0) | 854 (39) | 827 (60) | 815 (66) | 807 (74) | 798 (76) | 794 (82) | | 784 (88) | 689 |

| Number at risk (number censored) | | | | | | | | |  |  |
| --- | --- | --- | --- | --- | --- | --- | --- | --- | --- | --- |
| SP | 899 (0) | 833 (43) | 798 (61) | 784 (69) | 768 (87) | 755 (91) | 748 (98) | 735 (106) | | 637 |
| EP | 906 (0) | 854 (25) | 827 (42) | 815 (45) | 807 (54) | 798 (59) | 794 (64) | 784 (66) | | 689 |


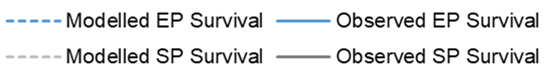


Figure A3: Estimated costs by resource use category per HIV-positive individual over the 48 week period for each of the strategies

Note: Pfx: Prophylactic medication; Non-Pfx: Non-prophylaxis medications; Clinic: Clinic visits; Hosp: Hospitalisations; EP: Enhanced-prophylaxis SP: Standard prophylaxis EPlessF: enhanced-prophylaxis less fluconazole SPplusF: Standard prophylaxis plus fluconazole. ART costs excluded from Figure.

Figure A4: Maximum prices for CrAg testing and CD4 testing for stratifying receipt of enhanced-prophylaxis

Table A1: Strategy details

| Strategy | CrAg test result | Initial 12 weeks on ART | Post 12 weeks in countries where isoniazid prophylaxis is standard of care | Post 12 weeks in countries where isoniazid prophylaxis is not standard of care |
| --- | --- | --- | --- | --- |
| Strategy 1- Standard prophylaxis | N/A | 160mg trimethoprim/800mg sulfamethoxazole daily | FDC of trimethoprim-sulfmethoxazole, isoniazid (300mg) and pyridoxine (25mg) daily | 160mg trimethoprim/800mg sulfamethoxazole daily |
| Strategy 2: Enhanced-prophylaxis (including universal fluconazole) | N/A | FDC of trimethoprim-sulfmethoxazole, isoniazid (300mg) and pyridoxine (25mg) daily, 100mg fluconazole daily, single-dose albendazole (400mg) and 5 days’ azithromycin (500mg daily). | FDC of trimethoprim-sulfmethoxazole, isoniazid (300mg) and pyridoxine (25mg) daily | 160mg trimethoprim/800mg sulfamethoxazole daily |
| Strategy 3: Standard prophylaxis plus universal fluconazole | N/a | 160mg trimethoprim/800mg sulfamethoxazole and 100mg fluconazole daily | FDC of trimethoprim-sulfmethoxazole, isoniazid (300mg) and pyridoxine (25mg) daily | 160mg trimethoprim/800mg sulfamethoxazole daily |
| Strategy 4: CrAg EP+ve SP-ve | +ve CrAg | FDC of trimethoprim-sulfmethoxazole, isoniazid (300mg) and pyridoxine (25mg) daily, 100mg fluconazole daily, single-dose albendazole (400mg) and 5 days’ azithromycin (500mg daily). | FDC of trimethoprim-sulfmethoxazole, isoniazid (300mg) and pyridoxine (25mg) daily | 160mg trimethoprim/800mg sulfamethoxazole daily |
|  | -ve CrAg | 160mg trimethoprim/800mg sulfamethoxazole daily | FDC of trimethoprim-sulfmethoxazole, isoniazid (300mg) and pyridoxine (25mg) daily | 160mg trimethoprim/800mg sulfamethoxazole daily |
| Strategy 5: CrAg EP+ve EplessF-ve | +ve CrAg | FDC of trimethoprim-sulfmethoxazole, isoniazid (300mg) and pyridoxine (25mg) daily, 100mg fluconazole daily, single-dose albendazole (400mg) and 5 days’ azithromycin (500mg daily). | FDC of trimethoprim-sulfmethoxazole, isoniazid (300mg) and pyridoxine (25mg) daily | 160mg trimethoprim/800mg sulfamethoxazole daily |
|  | -ve CrAg | FDC of trimethoprim-sulfmethoxazole, isoniazid (300mg) and pyridoxine (25mg) daily, single-dose albendazole (400mg) and 5 days’ azithromycin (500mg daily) | FDC of trimethoprim-sulfmethoxazole, isoniazid (300mg) and pyridoxine (25mg) daily | 160mg trimethoprim/800mg sulfamethoxazole daily |
| Strategy 6: CrAg SPplusF+ve SP-ve | +ve CrAg | 160mg trimethoprim/800mg sulfamethoxazole and 100mg fluconazole daily | FDC of trimethoprim-sulfmethoxazole, isoniazid (300mg) and pyridoxine (25mg) daily | 160mg trimethoprim/800mg sulfamethoxazole daily |
|  | -ve CrAg | 160mg trimethoprim/800mg sulfamethoxazole daily | FDC of trimethoprim-sulfmethoxazole, isoniazid (300mg) and pyridoxine (25mg) daily | 160mg trimethoprim/800mg sulfamethoxazole daily |

Note: EP=enhanced-prophylaxis, SP=standard-prophylaxis, EPlessF= enhanced-prophylaxis less fluconazole, SPplusF=standard-prophylaxis plus fluconazole.

Table A2: Patient-level covariate values by modelled CD4 bin

| - | **CD4 0-10** | **CD4 10-20** | **CD4 20-30** | **CD4 30-40** | **CD4 40-50** | **CD4 50-60** | **CD4 60-70** | **CD4 70-80** | **CD4 80-90** | **CD4 90-100** | **CD4 100-200*** |
| --- | --- | --- | --- | --- | --- | --- | --- | --- | --- | --- | --- |
| CrAg prevalence | 13.03% | 10.40% | 9.05% | 8.74% | 6.84% | 9.88% | 6.40% | 5.00% | 6.74% | 6.90% | 2.67% |
| Baseline CD4 | 6.10 | 14.93 | 25.38 | 35.40 | 45.32 | 55.28 | 65.76 | 75.21 | 85.93 | 94.51 | - |
| Age (years) | 34.47 | 34.49 | 36.68 | 35.88 | 37.89 | 37.38 | 35.82 | 36.21 | 37.14 | 39.80 | 39.80 |
| Sex (male %) | 50.95% | 54.85% | 55.92% | 53.14% | 54.74% | 54.55% | 54.40% | 45.39% | 42.70% | 63.79% | 63.79% |
| EQ-5D baseline** | 0.81 | 0.82 | 0.82 | 0.85 | 0.86 | 0.85 | 0.88 | 0.88 | 0.86 | 0.86 | 0.86 |

*The midpoint CD4 value was used for each bin in the CD4 100-200 range; CrAg prevalence (2.67%) was derived as 30.76923% [informed by Ford et al (2018)] of the observed CD4 0-100 REALITY prevalence; other covariates values were taken from the CD4 90-100 bin

**Base case baseline EQ-5D scoring where full-self reported health is assigned an EQ-5D score of 1

Table A3: Baseline CD4 distributions for each modelled HIV population

|  | **Baseline CD4 distributions** | | | | | | | | | |
| --- | --- | --- | --- | --- | --- | --- | --- | --- | --- | --- |
| **HIV population*** | **0-10** | **10-20** | **20-30** | **30-40** | **40-50** | **50-60** | **60-70** | **70-80** | **80-90** | **90-100** |
| CD4 0-100 | 15.05% | 17.11% | 12.07% | 11.84% | 10.87% | 9.44% | 7.15% | 8.07% | 5.09% | 3.32% |
|  | **100-110** | **110-120** | **120-130** | **130-140** | **140-150** | **150-160** | **160-170** | **170-180** | **180-190** | **190-200** |
| CD4 100-200 | 11.32% | 10.82% | 8.90% | 11.25% | 11.67% | 9.47% | 9.68% | 9.68% | 9.25% | 7.97% |
|  | **0-100** | | | | | **100-200** | | | | |
| CD4 0-200 | 51.14% | | | | | 48.86% | | | | |
|  | **0-100** | | | **100-200** | | | **200+** | | | |
| HIV+ | 16.80% | | | 16.10% | | | 67.10% | | | |

*CD4 0-100: Distribution informed by the REALITY trial; CD4 100-200: Distribution informed by the DART trial; CD4 0-200: Proportions in each respective advanced HIV population informed by Carmona et al (2018); Distribution across all HIV+ patients informed by Carmona et al (2018)

Table A4: Unit costs of resource use and average ART weekly costs ($)

| **Costs ($)** | **Zimbabwe** | **Uganda** | **Malawi** | **Kenya** | **Source** |
| --- | --- | --- | --- | --- | --- |
| Clinic visit | 1.082 | 1.067 | 0.606 | 1.572 | WHO Choice [1] |
| Daily hospitalisation cost | 5.333 | 5.299 | 2.504 | 8.196 | WHO Choice*  [1] |
| Average weekly cost of ART | 2.18 | 2.08 | 2.20 | 2.07 | International Drug Price Indicator Guide [2] and REALITY trial data |
| Concomitant medications | Various | Various | Various | Various | International Drug Price Indicator Guide [2] |

Note: 2016 prices. See REALITY main trial publication for different ART regimens which were based on nucleoside reverse transcriptase inhibitor combinations of tenofovir/lamivudine, zidovudine/lamivudine and abacavir/lamivudine with either efavirenz or nevirapine as the third drug.

* WHO-CHOICE are estimates of the hotel component of the hospital costs, i.e. excluding drugs and diagnostic tests but including costs such as personnel, capital and food.

Table A5: Cause-specific piecewise exponential regression competing risk model (REALITY data)

| - | **CM-specific**  Hazard ratio  (95% CI) | **Unknown-specific** Hazard ratio  (95% CI) | **TSO-specific**  Hazard ratio  (95% CI) |
| --- | --- | --- | --- |
| Enhanced prophylaxis | 0.29999**  (0.09779, 0.92029) | 0.61859**  (0.40272, 0.95016) | - |
| CrAg-positive | 42.62415**  (12.17535, 149.221) |  | 0.63460  (0.29522, 1.36413) |
| CD4 (per cell/mm^3^) | 0.97455**  (0.95106, 0.99861) | 0.98744**  (0.97937, 0.99558) | 0.99221**  (0.98567, 0.99879) |
| Weeks since initiation 8-24 | 0.30639**  (0.10267, 0.91429) | 0.37233**  (0.23563, 0.58832) | 0.22620**  (0.14659, 0.34903) |
| Weeks since initiation 24-48 | 0.13178**  (0.03567, 0.48682) | 0.10133**  (0.05360, 0.19156) | 0.11464**  (0.07033, 0.18684) |
| Baseline hazard | 0.00007**  (0.00002, 0.00026) | 0.00092**  (0.00060, 0.00141) | 0.00101**  (0.00072, 0.00141) |
| **AIC** | **198.633** | **1041.515** | **1332.913** |

Note: CM cryptococcal meningitis mortality; TSO tuberculosis, specific infections and other known mortality; CrAg: cryptococcal antigen positive at baseline; *p<0.1, **p<0.05

Table A6: Clinic visits and ART costing regressions (REALITY data)

|  | **Clinic visit costs**  (95% CI) | **ART costs**  (95% CI) |
| --- | --- | --- |
| Age (per year) | 0.00086**  (0.00012, 0.00159) | 0.00105**  (0.00005, 0.00205) |
| Country |  |  |
| Uganda | -0.11594**  (-0.13560, -0.09629) | -0.05096**  (-0.07630, -0.02561) |
| Malawi | -0.68964**  (-0.73172, -0.64756) | 0.00542  (-0.02658, 0.03741) |
| Kenya | 0.27968**  (0.26131, 0.29805) | -0.05436**  (-0.08423, -0.02449) |
| Weeks since initiation 12-24 | -0.63279**  (-0.66615, 0.59943) | - |
| Weeks since initiation 24-48 | -1.30852**  (-1.34937, -1.26767) | - |
| _cons† | -0.96490**  (-0.98260, -0.94719) | 0.78358**  (0.76579, 0.80137) |

8-24: 8-24 weeks post baseline; 24-48: 24-48 weeks post baseline; _cons: constant

GEE panel regression with a log transformation of the dependent variable (long link) and a Gaussian family form.

† the constant has the interpretation of a 36 year old individual from Zimbabwe for ART costs and the interpretation of a 36 year old individual from Zimbabwe 0-12 weeks post baseline for clinic visits (age is a centred covariate)

*p<0.1, **p<0.05

Table A7: Hospitalisation and concomitant medication regressions (REALITY data)

|  | **Other hospitalisations**  **model coefficients**  (95% CI) | | **Cryptococcal hospitalisations**  **model coefficients**  (95% CI) | | | **Concomitant drug costs**  **model coefficients**  (95% CI) | | |
| --- | --- | --- | --- | --- | --- | --- | --- | --- |
|  | **Part 1^ꝉ^** | **Part 2^‡^** | **Part 1^ꝉ^** | **Part 2^‡^** | **Part 1^ꝉ^** | | **Part 2^‡^** |  |
| EP | 0.84356  (0.67404, 1.05573) | - | 0.55576 **  (0.32861, 0.93994) | - | - | | -0.15897*  (-0.33598, 0.01804) |  |
| CrAg | 0.43836**  (0.25244, 0.76120) | - | 99.21507**  (44.64673, 220.47820) | - | - | | 1.20250**  (0.87009, 1.53491) |  |
| EP*CrAg | - | - |  | - | - | | - |  |
| CD4 (per cell/mm^3^) | 0.99576**  (0.99182, 0.99971) | - | 0.97722**  (0.96545, 0.98913) | - | 0.99788*  (0.99549, 1.00027) | | -0.00371**  (-0.00665, -0.00077) |  |
| Sex | - | 0.07195**  (0.00720, 0.13670) | - | - | 0.77534**  (0.67440, 0.89139) | | - |  |
| Age (per year) | - | -0.00267  (-0.00609, 0.00074) | - | - | 1.01028**  (1.00342, 1.01718) | | -0.00986**  (-0.01914, -0.00057) |  |
| Country |  |  |  |  |  | |  |  |
| Uganda | 1.65119**  (1.25130, 2.17888) | 0.06200  (-0.02340, 0.14740) | - | 0.11674  (-0.06136, 0.29483) | 1.62611**  (1.37877, 1.91783) | | 0.47159**  (0.27915, 0.66403) |  |
| Malawi | 0.89145  (0.58155, 1.36650) | -0.75490**  (-1.00839, -0.50142) | - | -0.79143**  (-1.20931, -0.37355) | 0.27162**  (0.18975, 0.38881) | | 1.44046**  (0.93618, 1.94474) |  |
| Kenya | 1.13270  (0.80315, 1.59747) | 0.55517**  (0.46803, 0.64230) | - | 0.30837**  (0.029978, 0.58676) | 1.32668**  (1.09257, 1.61095) | | 0.82487**  (0.57833, 1.07141) |  |
| Time |  |  |  |  |  | |  |  |
| Weeks since initiation | 0.95874**  (0.95341, 0.96410) | - | - |  | - | | - |  |
| 2-4 | - | - | - |  | 0.98044  (0.87740, 1.09558) | | - |  |
| 4-8 | - | - | - |  | 0.53852**  (0.48455, 0.59850) | | - |  |
| 8-18 | - | - | 0.39792**  (0.25724, 0.61552) |  | 0.28531**  (0.25735, 0.31630) | | - |  |
| 18-48 | - | - | 0.12600**  (0.07545, 0.21041) |  | 0.18841**  (0.17009, 0.20870) | | - |  |
| Weeks prior to mortality |  |  |  |  |  | |  |  |
| 0-4 | 6.32757**  (4.79751, 8.34562) | 0.12886**  (0.04769, 0.21004) | 12.30492**  (6.78732, 22.30796) |  | 1.94164**  (1.55550, 2.42363) | | 0.49798**  (0.04279, 0.95318) |  |
| 4-8 | 3.24993**  (2.15663, 4.89746) | 0.09765  (-0.02474, 0.22005) | - |  | 1.41959**  (1.07149, 1.88078) | | - |  |
| 8-12 | 2.17281**  (1.21314, 3.89165) | - | - |  |  | | - |  |
| Constant term | 0.02805**  (0.02178, 0.03611) | 3.09090**  (3.01177, 3.17003) | 0.00045**  (0.00020, 0.00103) | 3.28335**  (3.13548, 3.43123) | 0.23494**  (0.20065, 0.27510) | | -1.08793**  (-1.25170, -0.92416) |  |

EP: enhanced prophylaxis; crag: cryptococcal antigen positive at baseline; 2-4: 2-4 weeks post baseline; 4-8: 4-8 weeks post baseline; 8-18 weeks post baseline; 18-48 weeks post baseline; week: continuous weekly covariate 0-48 weeks;

ꝉ Part 1: Weekly probability of a positive resource specific cost (e.g. probability that CM-specific hospitalisation costs are positive in a given week) modelled with GEE panel regression with a logit transformation of the dependent variable (logit link) and a binomial family form. Showing beta coefficients on the natural odds ratio scale.

‡ Part 2: Weekly category-specific cost conditional on the cost having been incurred (e.g. expected CM-specific hospitalisation cost conditional on having a CM-specific hospitalisation) modelled with GEE panel regression with a log transformation of the dependent variable (log link) and a Gaussian family form. Showing beta coefficients

† age and baseline CD4 are centred covariates with the constant interpretable as a Zimbabwe 36 year female (when sex is included) individual with a baseline CD4 count of 36 (when CD4 is included) on standard prophylaxis (when EP is included) who is CrAg –ve at baseline (when crag is included).

*p<0.1, **p<0.05

Table A8: HRQoL weights regression analyses (REALITY data)

|  | **Model**  (95% CI) |
| --- | --- |
| Enhanced prophylaxis | 0.00721  (-0.00185, 0.01627) |
| CD4 (per cell/mm^3^) | 0.00021**  (0.00006, 0.00036) |
| EQ-5D baseline | - |
| Age (per year) | -0.01027**  (-0.01798, -0.00256) |
| Country |  |
| Uganda | 0.00191  (-0.00920, 0.01301) |
| Malawi | 0.06176**  (0.04731, 0.07621) |
| Kenya | 0.05912**  (0.04609, 0.07215) |
| Time |  |
| Week 2-4 | 0.01660**  (0.00883, 0.02437) |
| Week 4-8 | 0.04024**  (0.03245, 0.04803) |
| Week 8-12 | 0.05346**  (0.04560, 0.06131) |
| Week 12-18 | 0.05756**  (0.04969, 0.06542) |
| Week 18-24 | 0.06397**  (0.05609, 0.07185) |
| Week 24-36 | 0.06786**  (0.05999, 0.07572) |
| Week 36-48 | 0.07155**  (0.06362, 0.07948) |
| Weeks prior to mortality |  |
| Weeks 0-4 | -0.35907**  (-0.38193, -0.33620) |
| Weeks 4-8 | -0.19342**  (-0.21751, -0.16933) |
| Weeks 8-12 | -0.16388**  (-0.19256, -0.13520) |
| Constant | 0.87605**  (0.86147, 0.89064) |

Random effects panel regression

Table A9: Base-case cost-effectiveness results by CD4 subgroup for base-case

|  | | | | **Incremental net health benefit (probability of being CE)** | | | **Incremental cost-effectiveness ratios** | |
| --- | --- | --- | --- | --- | --- | --- | --- | --- |
|  | **Costs (US$)** | **QALY** | **LY** | **K=$100** | **K=$300** | **K=$500** | **Cost per QALY** | **Cost per LY** |
| **100≤CD4 <200 cells/mm^3^** |  |  |  |  |  |  |  |  |
| Strategy 1: SP | $122.92 | 0.83802 | 0.88383 | **(1)** | **(0.441)** | **(0.162)** |  |  |
| Strategy 3: SPplusF | $125.32 | 0.83821 | 0.88402 | **-0.02382 (0)** | **-0.00781 (0)** | **-0.00461 (0)** | **Ext Dominated** | **Ext Dominated** |
| Strategy 2: EP | $126.10 | 0.84953 | 0.88889 | **-0.02035 (0)** | **0.00089 (0.559)** | **0.00514 (0.838)** | **$276.81** | **$628.66** |
| Strategy 6: CrAg SPplusF+ve SP-ve | $128.63 | 0.83812 | 0.88393 | **-0.05699 (0)** | **-0.01893 (0)** | **-0.01131 (0)** | **Dominated** | **Dominated** |
| Strategy 4: CrAg EP+ve SP-ve | $128.66 | 0.83843 | 0.88406 | **-0.05703 (0)** | **-0.01874 (0)** | **-0.01108 (0)** | **Dominated** | **Dominated** |
| Strategy 5: CrAg EP+ve EPlessF-ve | $129.21 | 0.84944 | 0.88881 | **-0.05148 (0)** | **-0.00955 (0)** | **-0.00116 (0)** | **Dominated** | **Dominated** |
| **CD4 <10 cells/mm^3^** |  |  |  |  |  |  |  |  |
| Strategy 1: SP | $123.29 | 0.75133 | 0.77926 | **(0.154)** | **(0)** | **(0)** |  |  |
| Strategy 3: SPplusF | $124.98 | 0.76597 | 0.79345 | **-0.00224 (0.002)** | **0.00901 (0.001)** | **0.01126(0.003)** | **Ext Dominated** | **Ext Dominated** |
| Strategy 2: EP | $126.55 | 0.79445 | 0.81532 | **0.01058 (0.844)** | **0.03228 (0.999)** | **0.03661(0.997)** | **$75.46** | **$90.22** |
| Strategy 6: CrAg SPplusF+ve SP-ve | $128.58 | 0.76379 | 0.79134 | **-0.04041 (0)** | **-0.00517 (0)** | **0.00188(0)** | **Dominated** | **Dominated** |
| Strategy 4: CrAg EP+ve SP-ve | $128.84 | 0.76738 | 0.79410 | **-0.0394 (0)** | **-0.00243 (0)** | **0.00496(0)** | **Dominated** | **Dominated** |
| Strategy 5: CrAg EP+ve EPlessF-ve | $129.94 | 0.79219 | 0.81315 | **-0.02564 (0)** | **0.01869 (0)** | **0.02755(0)** | **Dominated** | **Dominated** |
| **10≤CD4 <20 cells/mm^3^** |  |  |  |  |  |  |  |  |
| Strategy 1: SP | $122.85 | 0.76930 | 0.79534 | **(0.326)** | **(0)** | **(0)** |  |  |
| Strategy 3: SPplusF | $124.73 | 0.77939 | 0.80509 | **-0.00869 (0)** | **0.00383 (0.001)** | **0.00633(0.003)** | **Ext Dominated** | **Ext Dominated** |
| Strategy 2: EP | $126.14 | 0.80587 | 0.82491 | **0.00368 (0.674)** | **0.0256 (0.999)** | **0.02999(0.997)** | **$89.94** | **$111.20** |
| Strategy 6: CrAg SPplusF+ve SP-ve | $128.26 | 0.77757 | 0.80333 | **-0.04585 (0)** | **-0.00977 (0)** | **-0.00256(0)** | **Dominated** | **Dominated** |
| Strategy 4: CrAg EP+ve SP-ve | $128.46 | 0.78026 | 0.80535 | **-0.04511 (0)** | **-0.00773 (0)** | **-0.00026(0)** | **Dominated** | **Dominated** |
| Strategy 5: CrAg EP+ve EPlessF-ve | $129.47 | 0.80398 | 0.82311 | **-0.03151 (0)** | **0.01262 (0)** | **0.02144(0)** | **Dominated** | **Dominated** |
| **20≤CD4 <30 cells/mm^3^** |  |  |  |  |  |  |  |  |
| Strategy 1: SP | $122.87 | 0.76801 | 0.80993 | **(0.621)** | **(0.002)** | **(0)** |  |  |
| Strategy 3: SPplusF | $124.87 | 0.77501 | 0.81683 | **-0.01306 (0)** | **0.00032 (0)** | **0.00299(0.003)** | **Ext Dominated** | **Ext Dominated** |
| Strategy 2: EP | $126.16 | 0.79893 | 0.83445 | **-0.00199 (0.379)** | **0.01995 (0.998)** | **0.02434(0.997)** | **$106.44** | **Ext Dominated** |
| Strategy 6: CrAg SPplusF+ve SP-ve | $128.37 | 0.77361 | 0.81545 | **-0.04946 (0)** | **-0.01275 (0)** | **-0.00541(0)** | **Dominated** | **Dominated** |
| Strategy 4: CrAg EP+ve SP-ve | $128.53 | 0.77574 | 0.80535 | **-0.04895 (0)** | **-0.01116 (0)** | **-0.0036(0)** | **Dominated** | **Dominated** |
| Strategy 5: CrAg EP+ve EPlessF-ve | $129.45 | 0.79748 | 0.83304 | **-0.03641 (0)** | **0.00751 (0)** | **0.0163(0)** | **Dominated** | **Dominated** |
| **30≤CD4 <40 cells/mm^3^** |  |  |  |  |  |  |  |  |
| Strategy 1: SP | $122.85 | 0.78805 | 0.82130 | **(0.793)** | **(0.004)** | **(0)** |  |  |
| Strategy 3: SPplusF | $124.93 | 0.79351 | 0.82663 | **-0.0153 (0)** | **-0.00146 (0)** | **0.00131 (0.003)** | **Ext Dominated** | **Ext Dominated** |
| Strategy 2: EP | $126.10 | 0.81574 | 0.84237 | **-0.00476 (0.207)** | **0.01688 (0.996)** | **0.0212 (0.997)** | **$117.17** | **$154.04** |
| Strategy 6: CrAg SPplusF+ve SP-ve | $128.42 | 0.79240 | 0.82554 | **-0.05131 (0)** | **-0.0142 (0)** | **-0.00678 (0)** | **Dominated** | **Dominated** |
| Strategy 4: CrAg EP+ve SP-ve | $128.57 | 0.79433 | 0.82691 | **-0.05087 (0)** | **-0.01277 (0)** | **-0.00515 (0)** | **Dominated** | **Dominated** |
| Strategy 5: CrAg EP+ve EPlessF-ve | $129.39 | 0.81459 | 0.84126 | **-0.0388 (0)** | **0.00476 (0)** | **0.01348 (0)** | **Dominated** | **Dominated** |
| **40≤CD4 <50 cells/mm^3^** |  |  |  |  |  |  |  |  |
| Strategy 1: SP | $122.90 | 0.78347 | 0.83180 | **(0.957)** | **(0.01)** | **(0.004)** |  |  |
| Strategy 3: SPplusF | $125.09 | 0.78700 | 0.83530 | **-0.01831 (0)** | **-0.00375 (0)** | **-0.00084 (0)** | **Ext Dominated** | **Ext Dominated** |
| Strategy 2: EP | $126.17 | 0.80731 | 0.84937 | **-0.0088 (0.043)** | **0.01296 (0.99)** | **0.01731 (0.996)** | **$136.90** | **$185.72** |
| Strategy 6: CrAg SPplusF+ve SP-ve | $128.53 | 0.78612 | 0.83443 | **-0.05361 (0)** | **-0.0161 (0)** | **-0.0086 (0)** | **Dominated** | **Dominated** |
| Strategy 4: CrAg EP+ve SP-ve | $128.64 | 0.78751 | 0.83539 | **-0.05334 (0)** | **-0.01509 (0)** | **-0.00743 (0)** | **Dominated** | **Dominated** |
| Strategy 5: CrAg EP+ve EPlessF-ve | $129.41 | 0.80641 | 0.84849 | **-0.0421 (0)** | **0.00126 (0)** | **0.00993 (0)** | **Dominated** | **Dominated** |
| **50≤CD4 <60 cells/mm^3^** |  |  |  |  |  |  |  |  |
| Strategy 1: SP | $123.14 | 0.79717 | 0.83930 | **(0.951)** | **(0.012)** | **(0.004)** |  |  |
| Strategy 3: SPplusF | $125.28 | 0.80089 | 0.84296 | **-0.01776 (0)** | **-0.00344 (0)** | **-0.00058 (0)** | **Ext Dominated** | **Ext Dominated** |
| Strategy 2: EP | $126.30 | 0.81983 | 0.85554 | **-0.009 (0.049)** | **0.01211 (0.988)** | **0.01633 (0.996)** | **$139.73** | **$195.00** |
| Strategy 6: CrAg SPplusF+ve SP-ve | $128.80 | 0.80022 | 0.84230 | **-0.05354 (0)** | **-0.01581 (0)** | **-0.00827 (0)** | **Dominated** | **Dominated** |
| Strategy 4: CrAg EP+ve SP-ve | $128.95 | 0.80209 | 0.84354 | **-0.05322 (0)** | **-0.01446 (0)** | **-0.00671 (0)** | **Dominated** | **Dominated** |
| Strategy 5: CrAg EP+ve EPlessF-ve | $129.62 | 0.81914 | 0.85487 | **-0.04286 (0)** | **0.00036 (0)** | **0.00901 (0)** | **Dominated** | **Dominated** |
| **60≤CD4 <70 cells/mm^3^** |  |  |  |  |  |  |  |  |
| Strategy 1: SP | $122.88 | 0.82156 | 0.84811 | **(0.992)** | **(0.033)** | **(0.01)** |  |  |
| Strategy 3: SPplusF | $125.15 | 0.82367 | 0.85014 | **-0.02051 (0)** | **-0.00543 (0)** | **-0.00242 (0)** | **Ext Dominated** | **Ext Dominated** |
| Strategy 2: EP | $126.07 | 0.84144 | 0.86133 | **-0.01195 (0.008)** | **0.00927 (0.967)** | **0.01351 (0.99)** | **$160.10** | **$240.69** |
| Strategy 6: CrAg SPplusF+ve SP-ve | $128.57 | 0.82313 | 0.84962 | **-0.0553 (0)** | **-0.01739 (0)** | **-0.00981 (0)** | **Dominated** | **Dominated** |
| Strategy 4: CrAg EP+ve SP-ve | $128.67 | 0.82427 | 0.85034 | **-0.05512 (0)** | **-0.01657 (0)** | **-0.00886 (0)** | **Dominated** | **Dominated** |
| Strategy 5: CrAg EP+ve EPlessF-ve | $129.29 | 0.84089 | 0.86080 | **-0.04476 (0)** | **-0.00204 (0)** | **0.00651 (0)** | **Dominated** | **Dominated** |
| **70≤CD4 <80 cells/mm^3^** |  |  |  |  |  |  |  |  |
| Strategy 1: SP | $122.84 | 0.82654 | 0.85454 | **(0.998)** | **(0.068)** | **(0.015)** |  |  |
| Strategy 3: SPplusF | $125.15 | 0.82794 | 0.85589 | **-0.02169 (0)** | **-0.0063 (0)** | **-0.00322 (0)** | **Ext Dominated** | **Ext Dominated** |
| Strategy 2: EP | $126.03 | 0.84457 | 0.86596 | **-0.01385 (0.002)** | **0.0074 (0.932)** | **0.01166 (0.985)** | **$176.79** | **$279.01** |
| Strategy 6: CrAg SPplusF+ve SP-ve | $128.54 | 0.82750 | 0.85547 | **-0.056 (0)** | **-0.01803 (0)** | **-0.01043 (0)** | **Dominated** | **Dominated** |
| Strategy 4: CrAg EP+ve SP-ve | $128.61 | 0.82833 | 0.85597 | **-0.05589 (0)** | **-0.01744 (0)** | **-0.00975 (0)** | **Dominated** | **Dominated** |
| Strategy 5: CrAg EP+ve EPlessF-ve | $129.22 | 0.84413 | 0.86554 | **-0.04615 (0)** | **-0.00366 (0)** | **0.00484 (0)** | **Dominated** | **Dominated** |
| **80≤CD4 <90 cells/mm^3^** |  |  |  |  |  |  |  |  |
| Strategy 1: SP | $122.96 | 0.82649 | 0.86055 | **(0.999)** | **(0.101)** | **(0.019)** |  |  |
| Strategy 3: SPplusF | $125.26 | 0.82781 | 0.86184 | **-0.02171 (0)** | **-0.00636 (0)** | **-0.00329 (0)** | **Ext Dominated** | **Ext Dominated** |
| Strategy 2: EP | $126.12 | 0.84325 | 0.87079 | **-0.01484 (0.001)** | **0.00623 (0.899)** | **0.01045 (0.981)** | **$188.51** | **$308.70** |
| Strategy 6: CrAg SPplusF+ve SP-ve | $128.69 | 0.82748 | 0.86152 | **-0.05629 (0)** | **-0.0181 (0)** | **-0.01046 (0)** | **Dominated** | **Dominated** |
| Strategy 4: CrAg EP+ve SP-ve | $128.78 | 0.82853 | 0.86213 | **-0.05619 (0)** | **-0.01737 (0)** | **-0.0096 (0)** | **Dominated** | **Dominated** |
| Strategy 5: CrAg EP+ve EPlessF-ve | $129.35 | 0.84292 | 0.87047 | **-0.04746 (0)** | **-0.00486 (0)** | **0.00366 (0)** | **Dominated** | **Dominated** |
| **90≤CD4 <100 cells/mm^3^** |  |  |  |  |  |  |  |  |
| Strategy 1: SP | $123.26 | 0.80916 | 0.86503 | **(0.999)** | **(0.159)** | **(0.026)** |  |  |
| Strategy 3: SPplusF | $125.58 | 0.81022 | 0.86609 | **-0.02211 (0)** | **-0.00667 (0)** | **-0.00358 (0)** | **Ext Dominated** | **Ext Dominated** |
| Strategy 2: EP | $126.41 | 0.82467 | 0.87424 | **-0.01599 (0.001)** | **0.00501 (0.841)** | **0.0092 (0.974)** | **$203.13** | **$341.97** |
| Strategy 6: CrAg SPplusF+ve SP-ve | $129.01 | 0.80997 | 0.86583 | **-0.05664 (0)** | **-0.01834 (0)** | **-0.01069 (0)** | **Dominated** | **Dominated** |
| Strategy 4: CrAg EP+ve SP-ve | $129.10 | 0.81097 | 0.86640 | **-0.05659 (0)** | **-0.01766 (0)** | **-0.00987 (0)** | **Dominated** | **Dominated** |
| Strategy 5: CrAg EP+ve EPlessF-ve | $129.64 | 0.82441 | 0.87398 | **-0.04856 (0)** | **-0.00602 (0)** | **0.00248 (0)** | **Dominated** | **Dominated** |
| **100≤CD4 <110 cells/mm^3^** |  |  |  |  |  |  |  |  |
| Strategy 1: SP | $123.20 | 0.81618 | 0.87010 | **(1)** | **(0.256)** | **(0.054)** |  |  |
| Strategy 3: SPplusF | $125.58 | 0.81662 | 0.87054 | **-0.02331 (0)** | **-0.00747 (0)** | **-0.00431 (0)** | **Ext Dominated** | **Ext Dominated** |
| Strategy 2: EP | $126.36 | 0.83023 | 0.87782 | **-0.01756 (0)** | **0.00352 (0.744)** | **0.00773 (0.946)** | **$224.94** | **$409.48** |
| Strategy 6: CrAg SPplusF+ve SP-ve | $128.90 | 0.81642 | 0.87034 | **-0.05674 (0)** | **-0.01875 (0)** | **-0.01116 (0)** | **Dominated** | **Dominated** |
| Strategy 4: CrAg EP+ve SP-ve | $128.93 | 0.81678 | 0.87054 | **-0.05673 (0)** | **-0.01851 (0)** | **-0.01086 (0)** | **Dominated** | **Dominated** |
| Strategy 5: CrAg EP+ve EPlessF-ve | $129.48 | 0.83002 | 0.87762 | **-0.04894 (0)** | **-0.00708 (0)** | **0.00129 (0)** | **Dominated** | **Dominated** |
| **110≤CD4 <120 cells/mm^3^** |  |  |  |  |  |  |  |  |
| Strategy 1: SP | $123.17 | 0.82213 | 0.87419 | **(1)** | **(0.295)** | **(0.073)** |  |  |
| Strategy 3: SPplusF | $125.55 | 0.82248 | 0.87454 | **-0.02349 (0)** | **-0.0076 (0)** | **-0.00442 (0)** | **Ext Dominated** | **Ext Dominated** |
| Strategy 2: EP | $126.33 | 0.83539 | 0.88108 | **-0.01831 (0)** | **0.00273 (0.705)** | **0.00694 (0.927)** | **$238.13** | **$458.41** |
| Strategy 6: CrAg SPplusF+ve SP-ve | $128.87 | 0.82232 | 0.87438 | **-0.05683 (0)** | **-0.01882 (0)** | **-0.01122 (0)** | **Dominated** | **Dominated** |
| Strategy 4: CrAg EP+ve SP-ve | $128.91 | 0.82267 | 0.87455 | **-0.05684 (0)** | **-0.01859 (0)** | **-0.01094 (0)** | **Dominated** | **Dominated** |
| Strategy 5: CrAg EP+ve EPlessF-ve | $129.44 | 0.83523 | 0.88092 | **-0.04961 (0)** | **-0.00781 (0)** | **0.00055 (0)** | **Dominated** | **Dominated** |
| **120≤CD4 <130 cells/mm^3^** |  |  |  |  |  |  |  |  |
| Strategy 1: SP | $123.12 | 0.82769 | 0.87787 | **(1)** | **(0.347)** | **(0.097)** |  |  |
| Strategy 3: SPplusF | $125.51 | 0.82796 | 0.87814 | **-0.02363 (0)** | **-0.0077 (0)** | **-0.00451 (0)** | **Ext Dominated** | **Ext Dominated** |
| Strategy 2: EP | $126.28 | 0.84025 | 0.88403 | **-0.01902 (0)** | **0.00203 (0.653)** | **0.00624 (0.903)** | **$251.46** | **$512.91** |
| Strategy 6: CrAg SPplusF+ve SP-ve | $128.83 | 0.82784 | 0.87802 | **-0.0569 (0)** | **-0.01887 (0)** | **-0.01126 (0)** | **Dominated** | **Dominated** |
| Strategy 4: CrAg EP+ve SP-ve | $128.86 | 0.82817 | 0.87817 | **-0.05692 (0)** | **-0.01866 (0)** | **-0.011 (0)** | **Dominated** | **Dominated** |
| Strategy 5: CrAg EP+ve EPlessF-ve | $129.39 | 0.84012 | 0.88390 | **-0.05025 (0)** | **-0.00846 (0)** | **-0.00011 (0)** | **Dominated** | **Dominated** |
| **130≤CD4 <140 cells/mm^3^** |  |  |  |  |  |  |  |  |
| Strategy 1: SP | $123.06 | 0.83290 | 0.88118 | **(1)** | **(0.386)** | **(0.123)** |  |  |
| Strategy 3: SPplusF | $125.46 | 0.83311 | 0.88139 | **-0.02375 (0)** | **-0.00778 (0)** | **-0.00458 (0)** | **Ext Dominated** | **Ext Dominated** |
| Strategy 2: EP | $126.22 | 0.84484 | 0.88670 | **-0.01968 (0)** | **0.0014 (0.614)** | **0.00562 (0.877)** | **$264.83** | **$573.44** |
| Strategy 6: CrAg SPplusF+ve SP-ve | $128.77 | 0.83301 | 0.88130 | **-0.05697 (0)** | **-0.01891 (0)** | **-0.0113 (0)** | **Dominated** | **Dominated** |
| Strategy 4: CrAg EP+ve SP-ve | $128.80 | 0.83333 | 0.88144 | **-0.057 (0)** | **-0.01871 (0)** | **-0.01106 (0)** | **Dominated** | **Dominated** |
| Strategy 5: CrAg EP+ve EPlessF-ve | $129.33 | 0.84474 | 0.88660 | **-0.05085 (0)** | **-0.00906 (0)** | **-0.0007 (0)** | **Dominated** | **Dominated** |
| **140≤CD4 <150 cells/mm^3^** |  |  |  |  |  |  |  |  |
| Strategy 1: SP | $122.98 | 0.83779 | 0.88417 | **(1)** | **(0.431)** | **(0.154)** |  |  |
| Strategy 3: SPplusF | $125.38 | 0.83796 | 0.88433 | **-0.02386 (0)** | **-0.00784 (0)** | **-0.00464 (0)** | **Ext Dominated** | **Ext Dominated** |
| Strategy 2: EP | $126.15 | 0.84919 | 0.88912 | **-0.02031 (0)** | **0.00083 (0.569)** | **0.00506 (0.846)** | **$278.17** | **$640.46** |
| Strategy 6: CrAg SPplusF+ve SP-ve | $128.69 | 0.83788 | 0.88426 | **-0.05701 (0)** | **-0.01895 (0)** | **-0.01133 (0)** | **Dominated** | **Dominated** |
| Strategy 4: CrAg EP+ve SP-ve | $128.73 | 0.83818 | 0.88439 | **-0.05705 (0)** | **-0.01876 (0)** | **-0.0111 (0)** | **Dominated** | **Dominated** |
| Strategy 5: CrAg EP+ve EPlessF-ve | $129.26 | 0.84912 | 0.88905 | **-0.05143 (0)** | **-0.00959 (0)** | **-0.00123 (0)** | **Dominated** | **Dominated** |
| **150≤CD4 <160 cells/mm^3^** |  |  |  |  |  |  |  |  |
| Strategy 1: SP | $122.89 | 0.84241 | 0.88687 | **(1)** | **(0.481)** | **(0.178)** |  |  |
| Strategy 3: SPplusF | $125.30 | 0.84253 | 0.88699 | **-0.02394 (0)** | **-0.0079 (0)** | **-0.00469 (0)** | **Ext Dominated** | **Ext Dominated** |
| Strategy 2: EP | $126.08 | 0.85333 | 0.89133 | **-0.02091 (0)** | **0.00031 (0.519)** | **0.00456 (0.822)** | **$291.41** | **$714.48** |
| Strategy 6: CrAg SPplusF+ve SP-ve | $128.61 | 0.84247 | 0.88694 | **-0.05705 (0)** | **-0.01897 (0)** | **-0.01136 (0)** | **Dominated** | **Dominated** |
| Strategy 4: CrAg EP+ve SP-ve | $128.64 | 0.84276 | 0.88705 | **-0.0571 (0)** | **-0.0188 (0)** | **-0.01113 (0)** | **Dominated** | **Dominated** |
| Strategy 5: CrAg EP+ve EPlessF-ve | $129.18 | 0.85327 | 0.89127 | **-0.05198 (0)** | **-0.01008 (0)** | **-0.0017 (0)** | **Dominated** | **Dominated** |
| **160≤CD4 <170 cells/mm^3^** |  |  |  |  |  |  |  |  |
| Strategy 1: SP | $122.80 | 0.84677 | 0.88931 | **(1)** | **(0.512)** | **(0.21)** |  |  |
| Strategy 3: SPplusF | $125.21 | 0.84687 | 0.88941 | **-0.02402 (0)** | **-0.00794 (0)** | **-0.00472 (0)** | **Ext Dominated** | **Ext Dominated** |
| Strategy 2: EP | $125.99 | 0.85727 | 0.89333 | **-0.02147 (0)** | **-0.00016 (0.488)** | **0.00411 (0.79)** | **$304.48** | **$795.99** |
| Strategy 6: CrAg SPplusF+ve SP-ve | $128.51 | 0.84682 | 0.88936 | **-0.05709 (0)** | **-0.01899 (0)** | **-0.01137 (0)** | **Dominated** | **Dominated** |
| Strategy 4: CrAg EP+ve SP-ve | $128.55 | 0.84710 | 0.88947 | **-0.05715 (0)** | **-0.01883 (0)** | **-0.01116 (0)** | **Dominated** | **Dominated** |
| Strategy 5: CrAg EP+ve EPlessF-ve | $129.09 | 0.85722 | 0.89328 | **-0.05251 (0)** | **-0.01053 (0)** | **-0.00214 (0)** | **Dominated** | **Dominated** |
| **170≤CD4 <180 cells/mm^3^** |  |  |  |  |  |  |  |  |
| Strategy 1: SP | $122.69 | 0.85091 | 0.89153 | **(1)** | **(0.561)** | **(0.24)** |  |  |
| Strategy 3: SPplusF | $125.11 | 0.85099 | 0.89160 | **-0.02408 (0)** | **-0.00798 (0)** | **-0.00475 (0)** | **Ext Dominated** | **Ext Dominated** |
| Strategy 2: EP | $125.91 | 0.86104 | 0.89516 | **-0.02201 (0)** | **-0.00059 (0.439)** | **0.0037 (0.76)** | **$317.33** | **$885.51** |
| Strategy 6: CrAg SPplusF+ve SP-ve | $128.41 | 0.85095 | 0.89157 | **-0.05711 (0)** | **-0.01901 (0)** | **-0.01139 (0)** | **Dominated** | **Dominated** |
| Strategy 4: CrAg EP+ve SP-ve | $128.44 | 0.85122 | 0.89166 | **-0.05718 (0)** | **-0.01885 (0)** | **-0.01119 (0)** | **Dominated** | **Dominated** |
| Strategy 5: CrAg EP+ve EPlessF-ve | $129.00 | 0.86100 | 0.89512 | **-0.05301 (0)** | **-0.01094 (0)** | **-0.00253 (0)** | **Dominated** | **Dominated** |
| **180≤CD4 <190 cells/mm^3^** |  |  |  |  |  |  |  |  |
| Strategy 1: SP | $122.58 | 0.85485 | 0.89353 | **(1)** | **(0.598)** | **(0.263)** |  |  |
| Strategy 3: SPplusF | $125.00 | 0.85491 | 0.89359 | **-0.02413 (0)** | **-0.00801 (0)** | **-0.00478 (0)** | **Ext Dominated** | **Ext Dominated** |
| Strategy 2: EP | $125.81 | 0.86465 | 0.89682 | **-0.02252 (0)** | **-0.00098 (0.402)** | **0.00333 (0.737)** | **$329.90** | **$983.56** |
| Strategy 6: CrAg SPplusF+ve SP-ve | $128.30 | 0.85488 | 0.89357 | **-0.05714 (0)** | **-0.01902 (0)** | **-0.0114 (0)** | **Dominated** | **Dominated** |
| Strategy 4: CrAg EP+ve SP-ve | $128.33 | 0.85515 | 0.89365 | **-0.05721 (0)** | **-0.01888 (0)** | **-0.01121 (0)** | **Dominated** | **Dominated** |
| Strategy 5: CrAg EP+ve EPlessF-ve | $128.91 | 0.86462 | 0.89679 | **-0.05349 (0)** | **-0.01132 (0)** | **-0.00288 (0)** | **Dominated** | **Dominated** |
| **190≤CD4 <200 cells/mm^3^** |  |  |  |  |  |  |  |  |
| Strategy 1: SP | $122.47 | 0.85862 | 0.89536 | **(1)** | **(0.637)** | **(0.284)** |  |  |
| Strategy 3: SPplusF | $124.89 | 0.85866 | 0.89541 | **-0.02418 (0)** | **-0.00803 (0)** | **-0.0048 (0)** | **Ext Dominated** | **Ext Dominated** |
| Strategy 2: EP | $125.72 | 0.86812 | 0.89834 | **-0.02301 (0)** | **-0.00134 (0.363)** | **0.003 (0.716)** | **$342.15** | **$1,090.65** |
| Strategy 6: CrAg SPplusF+ve SP-ve | $128.19 | 0.85864 | 0.89538 | **-0.05715 (0)** | **-0.01903 (0)** | **-0.01141 (0)** | **Dominated** | **Dominated** |
| Strategy 4: CrAg EP+ve SP-ve | $128.22 | 0.85890 | 0.89546 | **-0.05724 (0)** | **-0.01889 (0)** | **-0.01122 (0)** | **Dominated** | **Dominated** |
| Strategy 5: CrAg EP+ve EPlessF-ve | $128.81 | 0.86810 | 0.89832 | **-0.05395 (0)** | **-0.01166 (0)** | **-0.0032 (0)** | **Dominated** | **Dominated** |

Note: CE cost-effective; QALY quality adjusted life year; LY life-year; EP=enhanced-prophylaxis, SP=standard-prophylaxis, EPlessF= enhanced-prophylaxis less fluconazole, SPplusF=standard-prophylaxis plus fluconazole, Ext Dominated=extendedly dominated

Table A10: Scenario analyses CD4 0-100 cells/mm^3^

| **CD4 <100 cells/mm^3^** | | | | **Incremental net health benefit** | | | **Incremental cost-effectiveness ratios** | |
| --- | --- | --- | --- | --- | --- | --- | --- | --- |
|  | **Costs (US$)** | **QALY** | **LY** | **K=$100** | **K=$300** | **K=$500** | **Cost per QALY** | **Cost per LY** |
| **Country-specific drug costs** |  | | | | | | | |
| Strategy 1: SP | $125.98 | 0.78716 | 0.82176 |  |  |  |  |  |
| Strategy 6: CrAg SPplusF+ve SP-ve | $132.00 | 0.79251 | 0.82697 | **-0.05487** | **-0.01472** | **-0.00669** | **Ext Dominated** | **Ext Dominated** |
| Strategy 4: CrAg EP+ve SP-ve | $133.00 | 0.79454 | 0.82842 | **-0.06290** | **-0.01605** | **-0.00668** | **Ext Dominated** | **Ext Dominated** |
| Strategy 3: SPplusF | $133.13 | 0.79372 | 0.82814 | **-0.06503** | **-0.01731** | **-0.00776** | **Dominated** | **Dominated** |
| Strategy 5: CrAg EP+ve EPlessF-ve | $142.23 | 0.81436 | 0.84232 | **-0.13535** | **-0.02698** | **-0.00531** | **$597.64** | **$790.52** |
| Strategy 2: EP | $143.76 | 0.81560 | 0.84352 | **-0.09453** | **-0.01612** | **-0.00044** | **$1,233.36** | **$1,278.95** |
| **Zimbabwe-specific drug costs** |  | | | | | | | |
| Strategy 1: SP | $124.81 | 0.78716 | 0.82176 |  |  |  |  |  |
| Strategy 6: CrAg SPplusF+ve SP-ve | $130.74 | 0.79251 | 0.82697 | **-0.05397** | **-0.01442** | **-0.00651** | **Ext Dominated** | **Ext Dominated** |
| Strategy 3: SPplusF | $131.05 | 0.79372 | 0.82814 | **-0.05586** | **-0.01425** | **-0.00593** | **$952.43** | **$978.81** |
| Strategy 4: CrAg EP+ve SP-ve | $133.25 | 0.79454 | 0.82842 | **-0.07706** | **-0.02077** | **-0.00951** | **Ext Dominated** | **Ext Dominated** |
| Strategy 5: CrAg EP+ve EPlessF-ve | $157.64 | 0.81436 | 0.84232 | **-0.30114** | **-0.08225** | **-0.03847** | **Ext Dominated** | **Ext Dominated** |
| Strategy 2: EP | $158.49 | 0.81560 | 0.84352 | **-0.25442** | **-0.06942** | **-0.03242** | **$1,253.88** | **$1,784.08** |
| **Uganda-specific drug costs** |  | | | | | | | |
| Strategy 1: SP | $126.71 | 0.78716 | 0.82176 |  |  |  |  |  |
| Strategy 3: SPplusF | $130.16 | 0.79372 | 0.82814 | **-0.02796** | **-0.00495** | **-0.00035** | **Ext Dominated** | **Ext Dominated** |
| Strategy 6: CrAg SPplusF+ve SP-ve | $132.41 | 0.79251 | 0.82697 | **-0.05162** | **-0.01364** | **-0.00604** | **Dominated** | **Dominated** |
| Strategy 4: CrAg EP+ve SP-ve | $132.78 | 0.79454 | 0.82842 | **-0.05334** | **-0.01286** | **-0.00477** | **Ext Dominated** | **Ext Dominated** |
| Strategy 2: EP | $133.76 | 0.81560 | 0.84352 | **-0.04208** | **0.00493** | **0.01433** | **$247.99** | **$324.13** |
| Strategy 5: CrAg EP+ve EPlessF-ve | $135.54 | 0.81436 | 0.84232 | **-0.03315** | **0.00271** | **0.00989** | **Dominated** | **Dominated** |
| **Malawi-specific drug costs** |  | | | | | | | |
| Strategy 1: SP | $123.72 | 0.78716 | 0.82176 |  |  |  |  |  |
| Strategy 6: CrAg SPplusF+ve SP-ve | $131.29 | 0.79251 | 0.82697 | **-0.07035** | **-0.01988** | **-0.00979** | **Ext Dominated** | **Ext Dominated** |
| Strategy 4: CrAg EP+ve SP-ve | $131.80 | 0.79454 | 0.82842 | **-0.07338** | **-0.01954** | **-0.00877** | **Ext Dominated** | **Ext Dominated** |
| Strategy 5: CrAg EP+ve EPlessF-ve | $134.32 | 0.81436 | 0.84232 | **-0.07879** | **-0.00813** | **0.00600** | **$389.70** | **$515.47** |
| Strategy 3: SPplusF | $148.42 | 0.79372 | 0.82814 | **-0.24040** | **-0.07576** | **-0.04284** | **Dominated** | **Dominated** |
| Strategy 2: EP | $153.49 | 0.81560 | 0.84352 | **-0.19887** | **-0.05090** | **-0.02130** | **$15,456.14** | **$16,027.50** |
| **Kenya-specific drug costs** |  | | | | | | | |
| Strategy 1: SP | $126.28 | 0.78716 | 0.82176 |  |  |  |  |  |
| Strategy 3: SPplusF | $128.38 | 0.79372 | 0.82814 | **-0.01440** | **-0.00043** | **0.00236** | **Ext Dominated** | **Ext Dominated** |
| Strategy 2: EP | $130.90 | 0.81560 | 0.84352 | **-0.01770** | **0.01306** | **0.01921** | **$162.22** | **$212.03** |
| Strategy 6: CrAg SPplusF+ve SP-ve | $131.86 | 0.79251 | 0.82697 | **-0.05038** | **-0.01323** | **-0.00579** | **Dominated** | **Dominated** |
| Strategy 4: CrAg EP+ve SP-ve | $132.14 | 0.79454 | 0.82842 | **-0.05113** | **-0.01213** | **-0.00432** | **Dominated** | **Dominated** |
| Strategy 5: CrAg EP+ve EPlessF-ve | $134.18 | 0.81436 | 0.84232 | **-0.03735** | **0.00131** | **0.00905** | **Dominated** | **Dominated** |
| **FDC costs $12 per year** |  | | | | | | | |
| Strategy 1: SP | $123.73 | 0.78716 | 0.82176 |  |  |  |  |  |
| Strategy 3: SPplusF | $124.96 | 0.79372 | 0.82814 | **-0.00573** | **0.00246** | **0.00410** | **Ext Dominated** | **Ext Dominated** |
| Strategy 2: EP | $127.32 | 0.81560 | 0.84352 | **-0.00738** | **0.01650** | **0.02127** | **$125.95** | **$164.62** |
| Strategy 6: CrAg SPplusF+ve SP-ve | $129.21 | 0.79251 | 0.82697 | **-0.04945** | **-0.01292** | **-0.00561** | **Dominated** | **Dominated** |
| Strategy 4: CrAg EP+ve SP-ve | $129.48 | 0.79454 | 0.82842 | **-0.05004** | **-0.01176** | **-0.00411** | **Dominated** | **Dominated** |
| Strategy 5: CrAg EP+ve EPlessF-ve | $130.60 | 0.81436 | 0.84232 | **-0.03574** | **0.00185** | **0.00937** | **Dominated** | **Dominated** |
| **200mg fluconazole dosage** |  | | | | | | | |
| Strategy 1: SP | $122.90 | 0.78716 | 0.82176 |  |  |  |  |  |
| Strategy 3: SPplusF | $127.35 | 0.79372 | 0.82814 | **-0.03792** | **-0.00827** | **-0.00234** | **Ext Dominated** | **Ext Dominated** |
| Strategy 6: CrAg SPplusF+ve SP-ve | $128.66 | 0.79251 | 0.82697 | **-0.05226** | **-0.01385** | **-0.00617** | **Dominated** | **Dominated** |
| Strategy 2: EP | $128.69 | 0.81560 | 0.84352 | **-0.02943** | **0.00915** | **0.01686** | **$203.49** | **$265.97** |
| Strategy 4: CrAg EP+ve SP-ve | $128.83 | 0.79454 | 0.82842 | **-0.05195** | **-0.01240** | **-0.00449** | **Dominated** | **Dominated** |
| Strategy 5: CrAg EP+ve EPlessF-ve | $129.66 | 0.81436 | 0.84232 | **-0.00246** | **0.01294** | **0.01602** | **Dominated** | **Dominated** |
| **Full-health EQ-5D score 0.9** |  | | | | | | | |
| Strategy 1: SP | $122.90 | 0.71475 | 0.82176 |  |  |  |  |  |
| Strategy 3: SPplusF | $124.96 | 0.72064 | 0.82814 | **-0.01475** | **-0.00099** | **0.00177** | **Ext Dominated** | **Ext Dominated** |
| Strategy 2: EP | $126.14 | 0.73984 | 0.84352 | **-0.00735** | **0.01428** | **0.01861** | **$129.28** | **$149.13** |
| Strategy 6: CrAg SPplusF+ve SP-ve | $128.45 | 0.71956 | 0.82697 | **-0.05067** | **-0.01368** | **-0.00628** | **Dominated** | **Dominated** |
| Strategy 4: CrAg EP+ve SP-ve | $128.60 | 0.72134 | 0.82842 | **-0.05047** | **-0.01243** | **-0.00482** | **Dominated** | **Dominated** |
| Strategy 5: CrAg EP+ve EPlessF-ve | $129.43 | 0.73873 | 0.84232 | **-0.02658** | **0.00320** | **0.00915** | **Dominated** | **Dominated** |
| **Alternate specification for CM survival** |  | | | | | | | |
| Strategy 1: SP | $122.90 | 0.78718 | 0.82178 |  |  |  |  |  |
| Strategy 3: SPplusF | $124.96 | 0.79371 | 0.82814 | **-0.01406** | **-0.00033** | **0.00241** | **Ext Dominated** | **Ext Dominated** |
| Strategy 2: EP | $126.14 | 0.81560 | 0.84352 | **-0.00397** | **0.01762** | **0.02194** | **$113.98** | **$149.01** |
| Strategy 6: CrAg SPplusF+ve SP-ve | $128.50 | 0.79297 | 0.82741 | **-0.05017** | **-0.01287** | **-0.00541** | **Dominated** | **Dominated** |
| Strategy 4: CrAg EP+ve SP-ve | $128.66 | 0.79500 | 0.82886 | **-0.04972** | **-0.01137** | **-0.00369** | **Dominated** | **Dominated** |
| Strategy 5: CrAg EP+ve EPlessF-ve | $129.48 | 0.81483 | 0.84277 | **-0.02410** | **0.00604** | **0.01207** | **Dominated** | **Dominated** |
| **WHO pre-emptive therapy \| CrAg +ve** |  | | | | | | | |
| Strategy 1: SP | $122.90 | 0.78716 | 0.82176 |  |  |  |  |  |
| Strategy 2: EP | $126.14 | 0.81560 | 0.84352 | **-0.00401** | **0.01762** | **0.02195** | **$114.09** | **$149.13** |
| CrAg SP & WHO+ve SP-ve | $128.39 | 0.79547 | 0.82983 | **-0.04663** | **-0.01000** | **-0.00268** | **Dominated** | **Dominated** |
| CrAg EP & WHO+ve SP-ve | $128.44 | 0.79721 | 0.83099 | **-0.04535** | **-0.00842** | **-0.00103** | **Dominated** | **Dominated** |
| Strategy 4: CrAg EP+ve SP-ve | $128.60 | 0.79454 | 0.82842 | **-0.04968** | **-0.01164** | **-0.00403** | **Dominated** | **Dominated** |
| CrAg EP & WHO+ve EPlessF-ve | $129.26 | 0.81703 | 0.84489 | **-0.03378** | **0.00865** | **0.01714** | **$2,177.73** | **$2,268.84** |

Note: CE cost-effective; QALY quality adjusted life year; LY life-year; EP=enhanced-prophylaxis, SP=standard-prophylaxis, EPlessF= enhanced-prophylaxis less fluconazole, WHO=World Health Organization recommended fluconazole regimen, SPplusF=standard-prophylaxis plus fluconazole, Ext Dominated=extendedly dominated

Table A11: Scenario analyses CD4 100-200 cells/mm^3^

| **100≤CD4 <200 cells/mm3** | | | | **Incremental net health benefit** | | | **Incremental cost-effectiveness ratios** | |
| --- | --- | --- | --- | --- | --- | --- | --- | --- |
|  | **Costs (US$)** | **QALY** | **LY** | **K=$100** | **K=$300** | **K=$500** | **Cost per QALY** | **Cost per LY** |
| **Country-specific drug costs** |  | | | | | | | |
| Strategy 1: SP | $126.23 | 0.84129 | 0.88715 |  |  |  |  |  |
| Strategy 6: CrAg SPplusF+ve SP-ve | $132.09 | 0.84139 | 0.88726 | **-0.05845** | **-0.01941** | **-0.01161** | **Ext Dominated** | **Ext Dominated** |
| Strategy 4: CrAg EP+ve SP-ve | $132.37 | 0.84168 | 0.88737 | **-0.06102** | **-0.02008** | **-0.01189** | **Ext Dominated** | **Ext Dominated** |
| Strategy 3: SPplusF | $133.87 | 0.84148 | 0.88734 | **-0.07624** | **-0.02529** | **-0.01509** | **Dominated** | **Dominated** |
| Strategy 5: CrAg EP+ve EPlessF-ve | $141.87 | 0.85212 | 0.89150 | **-0.14557** | **-0.04131** | **-0.02045** | **$1,444.35** | **$5,811.65** |
| Strategy 2: EP | $144.05 | 0.85221 | 0.89159 | **-0.10886** | **-0.02908** | **-0.01312** | **$24,214.40** | **$24,645.88** |
| **Zimbabwe-specific drug costs** |  | | | | | | | |
| Strategy 1: SP | $124.94 | 0.84129 | 0.88715 |  |  |  |  |  |
| Strategy 6: CrAg SPplusF+ve SP-ve | $130.77 | 0.84139 | 0.88726 | **-0.05820** | **-0.01933** | **-0.01156** | **Ext Dominated** | **Ext Dominated** |
| Strategy 4: CrAg EP+ve SP-ve | $131.51 | 0.84168 | 0.88737 | **-0.06529** | **-0.02150** | **-0.01274** | **Ext Dominated** | **Ext Dominated** |
| Strategy 3: SPplusF | $131.65 | 0.84148 | 0.88734 | **-0.06690** | **-0.02217** | **-0.01323** | **Dominated** | **Ext Dominated** |
| Strategy 5: CrAg EP+ve EPlessF-ve | $157.30 | 0.85212 | 0.89150 | **-0.31278** | **-0.09704** | **-0.05389** | **$2,988.40** | **$9,122.32** |
| Strategy 2: EP | $158.74 | 0.85221 | 0.89159 | **-0.26893** | **-0.08243** | **-0.04513** | **$16,016.47** | **$20,168.07** |
| **Uganda-specific drug costs** |  | | | | | | | |
| Strategy 1: SP | $127.03 | 0.84129 | 0.88715 |  |  |  |  |  |
| Strategy 3: SPplusF | $130.84 | 0.84148 | 0.88734 | **-0.03791** | **-0.01251** | **-0.00743** | **Ext Dominated** | **Ext Dominated** |
| Strategy 6: CrAg SPplusF+ve SP-ve | $132.79 | 0.84139 | 0.88726 | **-0.05742** | **-0.01907** | **-0.01140** | **Dominated** | **Dominated** |
| Strategy 4: CrAg EP+ve SP-ve | $132.88 | 0.84168 | 0.88737 | **-0.05812** | **-0.01911** | **-0.01131** | **Ext Dominated** | **Ext Dominated** |
| Strategy 2: EP | $134.00 | 0.85221 | 0.89159 | **-0.05874** | **-0.01230** | **-0.00301** | **$637.93** | **$1,569.25** |
| Strategy 5: CrAg EP+ve EPlessF-ve | $135.45 | 0.85212 | 0.89150 | **-0.03538** | **-0.00470** | **0.00143** | **Dominated** | **Dominated** |
| **Malawi-specific drug costs** |  | | | | | | | |
| Strategy 1: SP | $123.76 | 0.84129 | 0.88715 |  |  |  |  |  |
| Strategy 6: CrAg SPplusF+ve SP-ve | $130.10 | 0.84139 | 0.88726 | **-0.06330** | **-0.02103** | **-0.01258** | **Ext Dominated** | **Ext Dominated** |
| Strategy 4: CrAg EP+ve SP-ve | $130.24 | 0.84168 | 0.88737 | **-0.06439** | **-0.02120** | **-0.01256** | **Ext Dominated** | **Ext Dominated** |
| Strategy 5: CrAg EP+ve EPlessF-ve | $132.55 | 0.85212 | 0.89150 | **-0.07701** | **-0.01845** | **-0.00674** | **$811.20** | **$2,019.32** |
| Strategy 3: SPplusF | $149.53 | 0.84148 | 0.88734 | **-0.25748** | **-0.08570** | **-0.05134** | **Dominated** | **Dominated** |
| Strategy 2: EP | $154.14 | 0.85221 | 0.89159 | **-0.22953** | **-0.06930** | **-0.03725** | **$239,501.60** | **$243,719.22** |
| **Kenya-specific drug costs** |  | | | | | | | |
| Strategy 1: SP | $126.57 | 0.84129 | 0.88715 |  |  |  |  |  |
| Strategy 3: SPplusF | $128.98 | 0.84148 | 0.88734 | **-0.02395** | **-0.00785** | **-0.00464** | **Ext Dominated** | **Ext Dominated** |
| Strategy 2: EP | $131.06 | 0.85221 | 0.89159 | **-0.03396** | **-0.00404** | **0.00194** | **$411.01** | **$1,011.04** |
| Strategy 6: CrAg SPplusF+ve SP-ve | $132.29 | 0.84139 | 0.88726 | **-0.05705** | **-0.01895** | **-0.01133** | **Dominated** | **Dominated** |
| Strategy 4: CrAg EP+ve SP-ve | $132.36 | 0.84168 | 0.88737 | **-0.05745** | **-0.01889** | **-0.01118** | **Dominated** | **Dominated** |
| Strategy 5: CrAg EP+ve EPlessF-ve | $134.16 | 0.85212 | 0.89150 | **-0.04107** | **-0.00660** | **0.00029** | **Dominated** | **Dominated** |
| **FDC costs $12 per year** |  | | | | | | | |
| Strategy 1: SP | $123.79 | 0.84129 | 0.88715 |  |  |  |  |  |
| Strategy 3: SPplusF | $125.29 | 0.84148 | 0.88734 | **-0.01481** | **-0.00481** | **-0.00281** | **Ext Dominated** | **Ext Dominated** |
| Strategy 2: EP | $127.29 | 0.85221 | 0.89159 | **-0.02417** | **-0.00078** | **0.00390** | **$321.39** | **$790.59** |
| Strategy 6: CrAg SPplusF+ve SP-ve | $129.48 | 0.84139 | 0.88726 | **-0.05680** | **-0.01886** | **-0.01128** | **Dominated** | **Dominated** |
| Strategy 4: CrAg EP+ve SP-ve | $129.54 | 0.84168 | 0.88737 | **-0.05719** | **-0.01880** | **-0.01112** | **Dominated** | **Dominated** |
| Strategy 5: CrAg EP+ve EPlessF-ve | $130.39 | 0.85212 | 0.89150 | **-0.04043** | **-0.00639** | **0.00042** | **Dominated** | **Dominated** |
| **200mg fluconazole dosage** |  | | | | | | | |
| Strategy 1: SP | $122.87 | 0.84129 | 0.88715 |  |  |  |  |  |
| Strategy 3: SPplusF | $127.75 | 0.84148 | 0.88734 | **-0.04854** | **-0.01605** | **-0.00955** | **Ext Dominated** | **Ext Dominated** |
| Strategy 6: CrAg SPplusF+ve SP-ve | $128.65 | 0.84139 | 0.88726 | **-0.05770** | **-0.01916** | **-0.01146** | **Dominated** | **Dominated** |
| Strategy 2: EP | $128.68 | 0.85221 | 0.89159 | **-0.04712** | **-0.00843** | **-0.00069** | **$531.55** | **$1,307.56** |
| Strategy 4: CrAg EP+ve SP-ve | $128.69 | 0.84168 | 0.88737 | **-0.05780** | **-0.01900** | **-0.01125** | **Dominated** | **Dominated** |
| Strategy 5: CrAg EP+ve EPlessF-ve | $129.23 | 0.85212 | 0.89150 | **-0.00421** | **0.00569** | **0.00767** | **Dominated** | **Dominated** |
| **Full-health EQ-5D score 0.9** |  | | | | | | | |
| Strategy 1: SP | $122.87 | 0.77180 | 0.88715 |  |  |  |  |  |
| Strategy 3: SPplusF | $125.29 | 0.77198 | 0.88734 | **-0.02396** | **-0.00787** | **-0.00465** | **Ext Dominated** | **Ext Dominated** |
| Strategy 2: EP | $126.06 | 0.78114 | 0.89159 | **-0.02255** | **-0.00129** | **0.00296** | **$341.49** | **$718.56** |
| Strategy 6: CrAg SPplusF+ve SP-ve | $128.59 | 0.77190 | 0.88726 | **-0.05705** | **-0.01895** | **-0.01133** | **Dominated** | **Dominated** |
| Strategy 4: CrAg EP+ve SP-ve | $128.62 | 0.77215 | 0.88737 | **-0.05715** | **-0.01882** | **-0.01116** | **Dominated** | **Dominated** |
| Strategy 5: CrAg EP+ve EPlessF-ve | $129.16 | 0.78106 | 0.89150 | **-0.02966** | **-0.00383** | **0.00133** | **Dominated** | **Dominated** |
| **Alternate specification for CM survival** |  | | | | | | | |
| Strategy 1: SP | $122.87 | 0.84130 | 0.88717 |  |  |  |  |  |
| Strategy 3: SPplusF | $125.28 | 0.84147 | 0.88733 | **-0.02394** | **-0.00787** | **-0.00465** | **Ext Dominated** | **Ext Dominated** |
| Strategy 2: EP | $126.06 | 0.85219 | 0.89158 | **-0.02097** | **0.00027** | **0.00452** | **$292.51** | **$721.95** |
| Strategy 6: CrAg SPplusF+ve SP-ve | $128.59 | 0.84141 | 0.88728 | **-0.05705** | **-0.01894** | **-0.01132** | **Dominated** | **Dominated** |
| Strategy 4: CrAg EP+ve SP-ve | $128.62 | 0.84170 | 0.88739 | **-0.05710** | **-0.01877** | **-0.01110** | **Dominated** | **Dominated** |
| Strategy 5: CrAg EP+ve EPlessF-ve | $129.16 | 0.85214 | 0.89152 | **-0.02811** | **-0.00226** | **0.00291** | **Dominated** | **Dominated** |
| **WHO pre-emptive therapy \| CrAg +ve** |  | | | | | | | |
| Strategy 1: SP | $122.87 | 0.84129 | 0.88715 |  |  |  |  |  |
| Strategy 2: EP | $126.06 | 0.85221 | 0.89159 | **-0.02098** | **0.00029** | **0.00454** | **$292.11** | **$718.56** |
| Strategy 4: CrAg EP+ve SP-ve | $128.62 | 0.84168 | 0.88737 | **-0.05710** | **-0.01877** | **-0.01111** | **Dominated** | **Dominated** |
| CrAg EP & WHO+ve SP-ve | $128.82 | 0.84173 | 0.88742 | **-0.05906** | **-0.01939** | **-0.01146** | **Dominated** | **Dominated** |
| CrAg SP & WHO+ve SP-ve | $128.83 | 0.84144 | 0.88730 | **-0.05944** | **-0.01971** | **-0.01177** | **Dominated** | **Dominated** |
| CrAg EP & WHO+ve EPlessF-ve | $129.36 | 0.85216 | 0.89155 | **-0.05400** | **-0.01075** | **-0.00210** | **Dominated** | **Dominated** |

Note: CE cost-effective; QALY quality adjusted life year; LY life-year; EP=enhanced-prophylaxis, SP=standard-prophylaxis, EPlessF= enhanced-prophylaxis less fluconazole, WHO=World Health Organization recommended fluconazole regimen, SPplusF=standard-prophylaxis plus fluconazole, Ext Dominated=extendedly dominated.

Table A12: Scenario analyses all HIV-positive

| **All individuals with HIV, regardless of CD4 count** | | | | **Incremental net health benefit** | | | **Incremental cost-effectiveness ratios** | |
| --- | --- | --- | --- | --- | --- | --- | --- | --- |
|  | **Costs (US$)** | **QALY** | **LY** | **K=$100** | **K=$300** | **K=$500** | **Cost per QALY** | **Cost per LY** |
| **Country-specific drug costs** |  | | | | | | | |
| Strategy 1: SP | $48.24 | 0.88708 | 0.90027 |  |  |  |  |  |
| Strategy 6: CrAg SPplusF+ve SP-ve | $53.99 | 0.88799 | 0.90116 | **-0.05661** | **-0.01826** | **-0.01059** | **Ext Dominated** | **Ext Dominated** |
| Strategy 4: CrAg EP+ve SP-ve | $54.21 | 0.88838 | 0.90143 | **-0.05837** | **-0.01859** | **-0.01063** | **Ext Dominated** | **Ext Dominated** |
| Strategy 3: SPplusF | $59.07 | 0.88821 | 0.90137 | **-0.10711** | **-0.03495** | **-0.02052** | **Dominated** | **Dominated** |
| Strategy 5: CrAg EP+ve EPlessF-ve | $63.98 | 0.89339 | 0.90443 | **-0.15108** | **-0.04615** | **-0.02517** | **$2,493.23** | **$3,788.21** |
| Strategy 2: EP | $69.18 | 0.89361 | 0.90464 | **-0.14627** | **-0.04501** | **-0.02476** | **$23,343.79** | **$24,176.77** |
| **Zimbabwe-specific drug costs** |  | | | | | | | |
| Strategy 1: SP | $48.02 | 0.88708 | 0.90027 |  |  |  |  |  |
| Strategy 6: CrAg SPplusF+ve SP-ve | $53.75 | 0.88799 | 0.90116 | **-0.05641** | **-0.01819** | **-0.01055** | **Ext Dominated** | **Ext Dominated** |
| Strategy 4: CrAg EP+ve SP-ve | $54.29 | 0.88838 | 0.90143 | **-0.06144** | **-0.01961** | **-0.01125** | **$4,816.12** | **$5,439.73** |
| Strategy 3: SPplusF | $54.99 | 0.88821 | 0.90137 | **-0.06863** | **-0.02212** | **-0.01282** | **Dominated** | **Dominated** |
| Strategy 5: CrAg EP+ve EPlessF-ve | $79.84 | 0.89339 | 0.90443 | **-0.31195** | **-0.09978** | **-0.05734** | **$5,100.32** | **Ext Dominated** |
| Strategy 2: EP | $81.27 | 0.89361 | 0.90464 | **-0.26956** | **-0.08611** | **-0.04942** | **$6,392.15** | **$6,620.24** |
| **Uganda-specific drug costs** |  | | | | | | | |
| Strategy 1: SP | $51.89 | 0.88708 | 0.90027 |  |  |  |  |  |
| Strategy 3: SPplusF | $56.01 | 0.88821 | 0.90137 | **-0.04008** | **-0.01261** | **-0.00711** | **Ext Dominated** | **Ext Dominated** |
| Strategy 6: CrAg SPplusF+ve SP-ve | $57.57 | 0.88799 | 0.90116 | **-0.05593** | **-0.01803** | **-0.01045** | **Dominated** | **Dominated** |
| Strategy 4: CrAg EP+ve SP-ve | $57.65 | 0.88838 | 0.90143 | **-0.05630** | **-0.01790** | **-0.01022** | **Ext Dominated** | **Ext Dominated** |
| Strategy 2: EP | $60.72 | 0.89361 | 0.90464 | **-0.08186** | **-0.02293** | **-0.01114** | **$1,352.47** | **$2,022.72** |
| Strategy 5: CrAg EP+ve EPlessF-ve | $63.93 | 0.89339 | 0.90443 | **-0.07407** | **-0.02124** | **-0.01067** | **Dominated** | **Dominated** |
| **Malawi-specific drug costs** |  | | | | | | | |
| Strategy 1: SP | $42.74 | 0.88708 | 0.90027 |  |  |  |  |  |
| Strategy 6: CrAg SPplusF+ve SP-ve | $48.83 | 0.88799 | 0.90116 | **-0.05999** | **-0.01939** | **-0.01127** | **Ext Dominated** | **Ext Dominated** |
| Strategy 4: CrAg EP+ve SP-ve | $48.94 | 0.88838 | 0.90143 | **-0.06067** | **-0.01936** | **-0.01109** | **Ext Dominated** | **Ext Dominated** |
| Strategy 5: CrAg EP+ve EPlessF-ve | $55.65 | 0.89339 | 0.90443 | **-0.12274** | **-0.03671** | **-0.01950** | **$2,044.40** | **$3,106.25** |
| Strategy 3: SPplusF | $69.64 | 0.88821 | 0.90137 | **-0.26784** | **-0.08853** | **-0.05266** | **Dominated** | **Dominated** |
| Strategy 2: EP | $77.14 | 0.89361 | 0.90464 | **-0.27752** | **-0.08876** | **-0.05101** | **$96,472.26** | **$99,914.71** |
| **Kenya-specific drug costs** |  | | | | | | | |
| Strategy 1: SP | $51.23 | 0.88708 | 0.90027 |  |  |  |  |  |
| Strategy 3: SPplusF | $53.72 | 0.88821 | 0.90137 | **-0.02375** | **-0.00716** | **-0.00384** | **Ext Dominated** | **Ext Dominated** |
| Strategy 6: CrAg SPplusF+ve SP-ve | $56.89 | 0.88799 | 0.90116 | **-0.05563** | **-0.01793** | **-0.01039** | **Dominated** | **Dominated** |
| Strategy 4: CrAg EP+ve SP-ve | $56.94 | 0.88838 | 0.90143 | **-0.05582** | **-0.01774** | **-0.01012** | **Ext Dominated** | **Ext Dominated** |
| Strategy 2: EP | $57.56 | 0.89361 | 0.90464 | **-0.05678** | **-0.01457** | **-0.00613** | **$968.78** | **$1,448.88** |
| Strategy 5: CrAg EP+ve EPlessF-ve | $60.66 | 0.89339 | 0.90443 | **-0.06426** | **-0.01797** | **-0.00871** | **Dominated** | **Dominated** |
| **FDC costs $12 per year** |  | | | | | | | |
| Strategy 1: SP | $46.21 | 0.88708 | 0.90027 |  |  |  |  |  |
| Strategy 3: SPplusF | $48.41 | 0.88821 | 0.90137 | **-0.02082** | **-0.00619** | **-0.00326** | **Ext Dominated** | **Ext Dominated** |
| Strategy 2: EP | $51.59 | 0.89361 | 0.90464 | **-0.04727** | **-0.01140** | **-0.00423** | **$823.26** | **$1,231.24** |
| Strategy 6: CrAg SPplusF+ve SP-ve | $51.85 | 0.88799 | 0.90116 | **-0.05543** | **-0.01787** | **-0.01035** | **Dominated** | **Dominated** |
| Strategy 4: CrAg EP+ve SP-ve | $51.90 | 0.88838 | 0.90143 | **-0.05559** | **-0.01766** | **-0.01008** | **Dominated** | **Dominated** |
| Strategy 5: CrAg EP+ve EPlessF-ve | $54.69 | 0.89339 | 0.90443 | **-0.05768** | **-0.01577** | **-0.00739** | **Dominated** | **Dominated** |
| **200mg fluconazole dosage** |  | | | | | | | |
| Strategy 1: SP | $44.93 | 0.88708 | 0.90027 |  |  |  |  |  |
| Strategy 3: SPplusF | $48.21 | 0.88821 | 0.90137 | **-0.03166** | **-0.00980** | **-0.00543** | **Ext Dominated** | **Ext Dominated** |
| Strategy 2: EP | $50.50 | 0.89361 | 0.90464 | **-0.04915** | **-0.01203** | **-0.00460** | **$852.02** | **$1,274.27** |
| Strategy 6: CrAg SPplusF+ve SP-ve | $50.62 | 0.88799 | 0.90116 | **-0.05605** | **-0.01807** | **-0.01048** | **Dominated** | **Dominated** |
| Strategy 4: CrAg EP+ve SP-ve | $50.66 | 0.88838 | 0.90143 | **-0.05601** | **-0.01780** | **-0.01016** | **Dominated** | **Dominated** |
| Strategy 5: CrAg EP+ve EPlessF-ve | $52.80 | 0.89339 | 0.90443 | **-0.04074** | **-0.01013** | **-0.00400** | **Dominated** | **Dominated** |
| **Full-health EQ-5D score 0.9** |  | | | | | | | |
| Strategy 1: SP | $44.93 | 0.86372 | 0.90027 |  |  |  |  |  |
| Strategy 3: SPplusF | $47.41 | 0.86474 | 0.90137 | **-0.02381** | **-0.00726** | **-0.00395** | **Ext Dominated** | **Ext Dominated** |
| Strategy 2: EP | $49.65 | 0.86944 | 0.90464 | **-0.04148** | **-0.01001** | **-0.00372** | **$825.22** | **$1,080.21** |
| Strategy 6: CrAg SPplusF+ve SP-ve | $50.58 | 0.86455 | 0.90116 | **-0.05568** | **-0.01801** | **-0.01048** | **Dominated** | **Dominated** |
| Strategy 4: CrAg EP+ve SP-ve | $50.61 | 0.86489 | 0.90143 | **-0.05566** | **-0.01778** | **-0.01020** | **Dominated** | **Dominated** |
| Strategy 5: CrAg EP+ve EPlessF-ve | $52.75 | 0.86924 | 0.90443 | **-0.04889** | **-0.01330** | **-0.00618** | **Dominated** | **Dominated** |
| **Alternate specification for CM survival** |  | | | | | | | |
| Strategy 1: SP | $44.93 | 0.88708 | 0.90028 |  |  |  |  |  |
| Strategy 3: SPplusF | $47.41 | 0.88821 | 0.90137 | **-0.02369** | **-0.00715** | **-0.00384** | **Ext Dominated** | **Ext Dominated** |
| Strategy 2: EP | $49.65 | 0.89361 | 0.90464 | **-0.04066** | **-0.00920** | **-0.00291** | **$722.93** | **$1,081.79** |
| Strategy 6: CrAg SPplusF+ve SP-ve | $50.59 | 0.88807 | 0.90124 | **-0.05559** | **-0.01787** | **-0.01033** | **Dominated** | **Dominated** |
| Strategy 4: CrAg EP+ve SP-ve | $50.62 | 0.88846 | 0.90150 | **-0.05553** | **-0.01759** | **-0.01000** | **Dominated** | **Dominated** |
| Strategy 5: CrAg EP+ve EPlessF-ve | $52.76 | 0.89347 | 0.90451 | **-0.04822** | **-0.01257** | **-0.00543** | **Dominated** | **Dominated** |
| **WHO pre-emptive therapy \| CrAg +ve** |  | | | | | | | |
| Strategy 1: SP | $44.93 | 0.88708 | 0.90027 |  |  |  |  |  |
| CrAg SP & WHO+ve SP-ve | $47.97 | 0.88850 | 0.90165 | **-0.02903** | **-0.00873** | **-0.00467** | **Ext Dominated** | **Ext Dominated** |
| Strategy 2: EP | $49.65 | 0.89361 | 0.90464 | **-0.04067** | **-0.00920** | **-0.00291** | **$722.27** | **$1,080.21** |
| Strategy 4: CrAg EP+ve SP-ve | $50.61 | 0.88838 | 0.90143 | **-0.05552** | **-0.01764** | **-0.01006** | **Dominated** | **Dominated** |
| CrAg EP & WHO+ve SP-ve | $52.48 | 0.88883 | 0.90186 | **-0.07373** | **-0.02340** | **-0.01334** | **Dominated** | **Dominated** |
| CrAg EP & WHO+ve EPlessF-ve | $54.62 | 0.89384 | 0.90487 | **-0.09011** | **-0.02553** | **-0.01261** | **$21,260.50** | **$22,166.32** |

Note: CE cost-effective; QALY quality adjusted life year; LY life-year; EP=enhanced-prophylaxis, SP=standard-prophylaxis, EPlessF= enhanced-prophylaxis less fluconazole, WHO=World Health Organization recommended fluconazole regimen, Ext Dominated=extendedly dominated
