# Supplementary material for: The cost‐effectiveness of prophylaxis strategies for individuals with advanced HIV starting treatment in Africa
Source: J Int AIDS Soc. 2020 Mar 27;23(3):e25469. doi: 10.1002/jia2.25469 (PMC7099175; doi:10.1002/jia2.25469)
Supplement: Supplementary file 2 [file JIA2-23-e25469-s002.docx]

Supplementary Figures and Tables
